# Structure and Function of the *Campylobacter jejuni* Chromosome Replication Origin

**DOI:** 10.3389/fmicb.2018.01533

**Published:** 2018-07-12

**Authors:** Pawel Jaworski, Rafal Donczew, Thorsten Mielke, Christoph Weigel, Kerstin Stingl, Anna Zawilak-Pawlik

**Affiliations:** ^1^Department of Microbiology, Ludwik Hirszfeld Institute of Immunology and Experimental Therapy, Polish Academy of Sciences, Wrocław, Poland; ^2^Max Planck Institute for Molecular Genetics, Berlin, Germany; ^3^Department of Life Science Engineering, Fachbereich 2, HTW Berlin, Berlin, Germany; ^4^National Reference Laboratory for Campylobacter, Department of Biological Safety, Federal Institute for Risk Assessment, Berlin, Germany

**Keywords:** Epsilonproteobacteria Campylobacter jejuni, initiation of chromosome replication, *oriC*, DnaA, DnaA box, orisome

## Abstract

*Campylobacter jejuni* is the leading bacterial cause of foodborne infections worldwide. However, our understanding of its cell cycle is poor. We identified the probable *C. jejuni* origin of replication (*oriC*) – a key element for initiation of chromosome replication, which is also important for chromosome structure, maintenance and dynamics. The herein characterized *C. jejuni oriC* is monopartite and contains (i) the DnaA box cluster, (ii) the DnaA-dependent DNA unwinding element (DUE) and (iii) binding sites for regulatory proteins. The cluster of five DnaA boxes and the DUE were found in the *dnaA-dnaN* intergenic region. Binding of DnaA to this cluster of DnaA-boxes enabled unwinding of the DUE *in vitro*. However, it was not sufficient to sustain replication of minichromosomes, unless the cluster was extended by additional DnaA boxes located in the 3′ end of *dnaA*. This suggests, that *C. jejuni oriC* requires these boxes to initiate or to regulate replication of its chromosome. However, further detailed mutagenesis is required to confirm the role of these two boxes in initiation of *C. jejuni* chromosome replication and thus to confirm partial localization of *C. jejuni oriC* within a coding region, which has not been reported thus far for any bacterial *oriC*. *In vitro* DUE unwinding by DnaA was inhibited by Cj1509, an orphan response regulator and a homolog of HP1021, that has been previously shown to inhibit replication in *Helicobacter pylori*. Thus, Cj1509 might play a similar role as a regulator of *C. jejuni* chromosome replication. This is the first systematic analysis of chromosome replication initiation in *C. jejuni*, and we expect that these studies will provide a basis for future research examining the structure and dynamics of the *C. jejuni* chromosome, which will be crucial for understanding the pathogens’ life cycle and virulence.

## Introduction

*Campylobacter jejuni* is a Gram-negative, microaerophilic bacterium that belongs to the Epsilon class of Proteobacteria, which has recently been proposed to constitute a separate phylum, the Epsilonbacteryota (Parkhill et al., 2000; Eppinger et al., 2004; Waite et al., 2017). *C. jejuni* colonizes the intestine of diverse animal species in a commensal manner. However, in humans, *C. jejuni* often invades intestinal epithelial cells and causes acute bacterial gastroenteritis (O Cróinín and Backert, 2012). The infection is generally self-limiting, but complications can arise and may include autoimmune sequelae like reactive arthritis, irritable bowel syndrome and Guillain–Barré syndrome (Kaakoush et al., 2015). *C. jejuni* is isolated from environmental samples; however, the main source of *C. jejuni* infections is the handling and consumption of contaminated poultry meat. Survival in or colonization of diverse niches indicates that *C. jejuni*, although quite stress-sensitive, can resist varying environmental conditions such as low temperature or atmospheric oxygen concentrations (Murphy et al., 2006). Chromosome replication is one of the most vulnerable processes, which has to be highly regulated, because unexpected interruption of replication (e.g., under stress conditions) may be fatal for the bacterium. Hence, a molecular understanding of chromosome replication in *C. jejuni* and other pathogens may provide new reliable ways to combat infections.

To precisely and efficiently regulate chromosome replication, bacteria control the process at the very first step – the initiation. Generally, bacteria initiate replication at *oriC*, a single, unique site on the chromosome. Bacterial *oriCs* consists of one or more clusters of DnaA binding sites (DnaA boxes), the DNA unwinding element (DUE) and the binding sites for regulatory proteins called oriBPs – origin binding proteins (Wolański et al., 2014; Marczynski et al., 2015). Upon initiation, the initiator protein DnaA binds to DnaA boxes at *oriC* and assembles into a filament that can distort double-stranded (ds) DNA at the DUE (Duderstadt and Berger, 2013; Wolański et al., 2014; Leonard and Grimwade, 2015; Katayama et al., 2017). Subsequently, the open complex serves as a platform for the assembly of a multiprotein replication machinery, called the replisome, which will synthesize the nascent chromosome. *oriC* can be mono- or bipartite, i.e., DnaA boxes can be gathered into a single (*Escherichia coli, Mycobacterium tuberculosis*) or two clusters (*Bacillus subtilis, Helicobacter pylori*) located in intergenic regions, usually in the vicinity of the *dnaA* – *dnaN* locus (Briggs et al., 2012; Wolański et al., 2014). Typical DnaA boxes are non-palindromic, oriented (i.e., 3′–5′ directed) 9-mers with sequences similar to the “perfect” high-affinity R-type *E. coli* DnaA box 5′-TTWTNCACA-3′ (Schaper and Messer, 1995; Leonard and Grimwade, 2011; Wolański et al., 2014). However, in *E. coli* and *Caulobacter cresentus* low-affinity DnaA boxes were also identified, which differ by 3–4 nucleotides from R-type DnaA boxes (McGarry et al., 2004; Kawakami et al., 2005; Taylor et al., 2011). High-affinity DnaA boxes are bound by ATP- and ADP-DnaA, while low-affinity DnaA boxes are exclusively bound by ATP-DnaA. The DUE is located outside of the cluster, adjacent to the last DnaA box in the scaffold. The DUE region has usually a size of around 50 bps and is rich in thymines and adenines (an AT-rich region) (Rajewska et al., 2012), which lower the thermodynamic stability of the DUE and makes it helically unstable, even in the absence of DnaA (Kowalski and Eddy, 1989). It should be noted that, despite minor differences, the *oriC* region unwound either due to intrinsic instability of the helix or in the presence of DnaA was shown to be similar [e.g., *E. coli oriC* (Kowalski and Eddy, 1989; Hwang and Kornberg, 1992a) or *B. subtilis oriC* (Krause et al., 1997)]. It has recently been shown that in many bacteria the DUE region proximal to the DnaA-box cluster encodes a 5′-TAG-3′ motif, named DnaA-trio. This motif is required by DnaA to open DNA and to assemble on ssDNA (Richardson et al., 2016). The primary role of the last module, oriBPs binding site, which binds to proteins that control *oriC* activity, is to efficiently transmit feedback information from the cell and/or environment to the *oriC* to rapidly adjust the replication rate (Wolański et al., 2014; Marczynski et al., 2015). oriBPs binding sequences can overlap with DnaA boxes or be located within the DUE or elsewhere within the *oriC*. They bind different classes of proteins, such as nucleoid-associated proteins (NAPs, e.g., *E. coli* IHF, Fis, SeqA) [(Waldminghaus and Skarstad, 2009; Wolański et al., 2014; Leonard and Grimwade, 2015) and references herein] or response regulators of two component systems (e.g., *E. coli* ArcA, *B. subtilis* Spo0A, *H. pylori* HP1021, *M. tuberculosis* MtrA) (Lee et al., 2001; Castilla-Llorente et al., 2006; Donczew et al., 2015; Purushotham et al., 2015). Thus, the oriBPs binding modules are highly diverse, both in structure and species specificity.

*oriCs* of four Epsilonproteobacteria have been identified to date (Donczew et al., 2012; Jaworski et al., 2016). The Epsilonproteobacterial origins typically co-localize with *ruvC-dnaA-dnaN*, with the exception of Helicobacteraceae species, in which this gene order is not conserved (e.g., *H. pylori dnaA* is located between *punB* and *comH*). They likely constitute bipartite origins, with clusters of DnaA boxes localized upstream (*oriC*1) and downstream (*oriC2*) of *dnaA*; DNA unwinding was shown to occur in *oriC2* (Donczew et al., 2012; Jaworski et al., 2016). The typical Epsilonproteobacterial 9-mer DnaA box consists of the core nucleotide sequence 5′-TTCAC-3′ (4–8 nt of a 9-mer), with the 5th residue strictly conserved. This specific DnaA box sequence, together with the significant changes in the DNA-binding motif of corresponding DnaAs, determines the unique molecular mechanism of the DnaA-DNA interaction (Jaworski et al., 2016). There are two known regulators of *H. pylori* chromosome replication: HobA and HP1021. HobA, a homolog of *E. coli* DiaA (Keyamura et al., 2007; Natrajan et al., 2007), binds to DnaA and controls its oligomerization upon *oriC* binding (Zawilak-Pawlik et al., 2007, 2011), while HP1021 binds to *H. pylori oriC* to preclude DnaA binding to DnaA boxes and inhibit DNA unwinding at *oriC* (Donczew et al., 2015). Homologs of HP1021 and HobA are found in other Epsilonproteobacteria, including *C. jejuni* (Schär et al., 2005; Zawilak-Pawlik et al., 2011).

In this work, we identified and characterized the probable *C. jejuni oriC.* The *C. jejuni oriC* is most likely monopartite with the initial unwinding region located between *dnaA* and *dnaN*. We call this region DnaA-dependent DNA unwinding element (DUE), although, unlike in *E. coli* or *B. subtilis* DUE (Kowalski and Eddy, 1989; Krause et al., 1997), formal proof of protein-independent instability was not conducted. However, this region is AT rich, it is predicted to be helically unstable and it is unwound by DnaA. The *dnaA–dnaN* intergenic region contains a cluster of five DnaA binding sites which enable DnaA to build up a complex capable of DUE unwinding *in vitro*. However, for self-replication of minichromosomes in *C. jejuni* an additional 3′ end of *dnaA* comprising further DnaA binding sites are essential. Thus, *C. jejuni oriC* might be the first example of a bacterial origin that is partially located within a coding region, however, further studies are required to confirm the essentiality of these two DnaA boxes for initiation of *C. jejuni* chromosome replication. There are numerous DnaA binding sites located in the vicinity of *oriC*, mainly within the *dnaA* gene, which might play regulatory roles in controlling the initiation of complex assembly. We also identified Cj1509 (*C. jejuni* 81116 or Cj1608 in *C. jejuni* 11168), a homolog of *H. pylori* HP1021 (Schär et al., 2005). Here, we show that Cj1509 binds to *C. jejuni oriC* and inhibits *oriC* unwinding at the DUE. We speculate that these orphan regulators are regulating chromosome replication in Epsilonproteobacteria in response to as yet unknown signaling pathways.

## Materials and Methods

### Materials, Strains and Culture Conditions

The plasmids, proteins and bacterial strains used in this work are listed in Supplementary Table S1. The oligonucleotide sequences are presented in Supplementary Table S2. *C. jejuni* 81116 genomic DNA was used as a template to amplify DNA fragments for cloning. *E. coli* was grown at 37°C on solid or liquid Luria-Bertani medium supplemented with 100 μg ml^-1^ ampicillin or 25 μg ml^-1^ kanamycin where necessary. *C. jejuni* was cultivated at 37°C or 42°C under microaerophilic conditions on Columbia Blood (CB) Agar (CM0331, Oxoid) or Brain Heart Infusion (BHI) Broth (CM1135, Oxoid) supplemented with trimethoprim and polymyxin B at final concentrations of 10 μg ml^-1^ and 2.5 U ml^-1^, respectively; colistin (10 μg ml^-1^) and kanamycin (25 μg ml^-1^) were added when necessary.

### Minichromosome Maintenance

**FIGURE 1:**
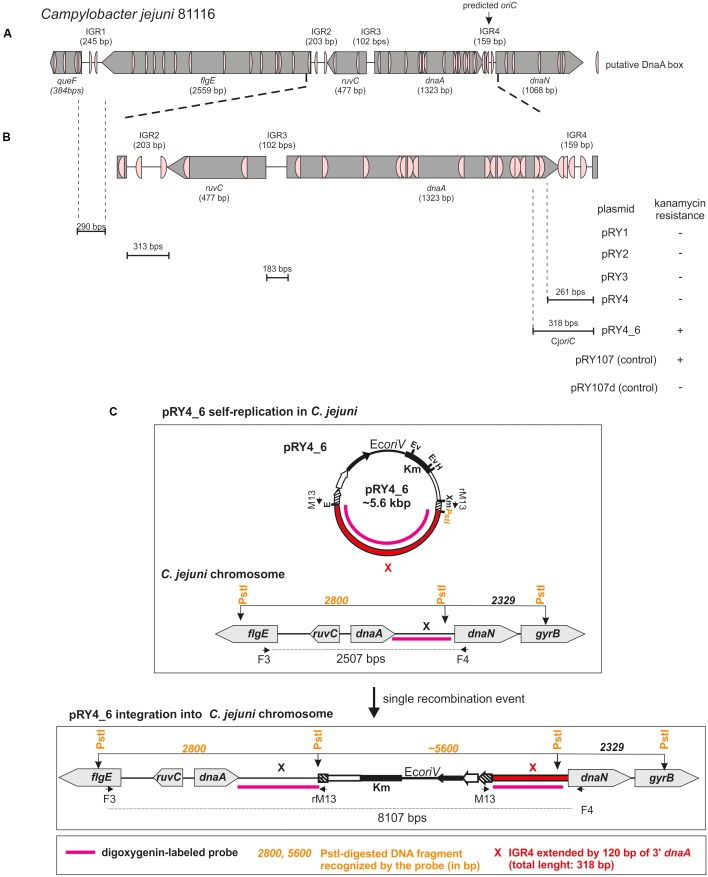
Identification of *Campylobacter jejuni oriC*. **(A)** Schematic presentation of a chromosomal region containing predicted *oriC* (Gao et al., 2013). The genes and intergenic regions (IGR) are drawn to scale with sizes indicated in brackets. Putative DnaA boxes identified according to the Epsilonproteobacterial DnaA box consensus sequence (Jaworski et al., 2016)) are marked. **(B)** Schematic presentation of the results of *oriC*-plasmid maintenance analysis. IGR regions cloned into non-replicating pRY107d are presented below the chromosomal scheme. Plasmids were introduced into *C. jejuni* cells by conjugation (see section “Materials and Methods”). Plasmids conferring *C. jejuni* resistance to kanamycin are marked by “+,” while “–“ denotes – no growth of *C. jejuni* conjugates on kanamycin. **(C)** Possible scenarios of *oriC* plasmids’ fate in *C. jejuni*. Plasmids containing *oriC* can self-replicate or incorporate into *C. jejuni* chromosome via single crossing over. In each case it will confer *C. jejuni* resistance to kanamycin.

pRY107d was prepared by HindIII digestion of pRY107 and religation. The IGR regions were amplified by PCR using specific primer pairs (Supplementary Figure S1 and Supplementary Table S2) and inserted between the EcoRI and PstI restriction sites of pRY107d to generate pRY_X (X-respective IGR region, see **Figure 1B**, Supplementary Table S1 and Supplementary Figure S2A). The pRY plasmids were introduced into *C. jejuni* 81–176 using a conjugation protocol that has been described previously (Van Vliet et al., 1998; Zeng et al., 2015), with minor modifications. *C. jejuni* recipient cells were grown overnight on CB agar plates at 37°C under microaerophilic conditions and subsequently harvested using an inoculating loop and 2 ml of BHI broth pre-warmed to 50°C, diluted to OD600 = 1 and incubated at 50°C for 30 min. Washed *E. coli* S17-1 donor cells were resuspended in 0.5 ml of *C. jejuni* cells. The mixture was concentrated by centrifugation to 100 μl and placed on a CB agar plate without antibiotics. After a 5-h incubation at 42°C under microaerophilic conditions, the cells were harvested with 1 ml of BHI, centrifuged, resuspended in 100 μl of BHI and spread on CB plates supplemented with trimethoprim, polymyxin B, colistin and kanamycin. The plates were incubated at 42°C under microaerophilic conditions for 2–4 days. Four colonies of each conjugation were streaked on CB agar plates supplemented with selective antibiotics, incubated for 2 days and harvested. Genomic DNA was purified and used as a template for PCR with the following primer pairs: B4-B5, F1-F2, M13-rM13, and F3-F4 or in Southern blot analysis. Southern blot was performed as described (Sambrook and Russell, 2001). Briefly 10–20 μg of *C. jejuni* genomic DNA and 10 ng of a control plasmid DNA isolated from *E. coli*, undigested or digested with appropriate restriction enzymes, were resolved in 1% agarose gel. DNA was transferred onto a nylon membrane and incubated at 58°C with digoxigenin-labeled DNA probe (314 bp DNA, amplified with primers A3–A4). Southern blot was developed by colorimetric reaction using anti-digoxigenin antibody (Anti-Digoxigenin-AP, Fab fragments, Roche). The presence of the self-replicating pRY4_6 plasmid in *C. jejuni* was additionally analyzed by transformation of *E. coli* TOP10 competent cells with genomic DNA isolated from *C. jejuni* pRY4_6 conjugants. Plasmid DNA was purified from *E. coli* colonies and analyzed by digestion with EcoRI and PstI.

### Protein Expression and Purification

The *dnaA* gene was amplified by PCR using primer pairs B1–B2 and B1–B3 and inserted between the BamHI-XhoI restriction sites of pET28a(+) and pT21Strep, to generate pET28CjDnaA and pET21CjDnaA, respectively. pET21Strep is pET21b(+) derivative modified by removal of T7tag sequence and insertion of the Strep-tag sequence (for details see Supplementary Table S1 and Supplementary Figure S4A). 6HisCjDnaA (N-terminal His-tagged) and StrepCjDnaA (C-terminal Strep-tagged) (Supplementary Figure S4B) were expressed and purified as described previously (Zawilak-Pawlik et al., 2006) or according to Strep-Tactin manufacturer’s protocol (IBA Lifesciences). The activities of recombinant His-tagged and Strep-tagged proteins were similar to other Epsilonproteobacterial DnaAs, which was confirmed by using similar experimental conditions as previously published. In particular, in the P1 nuclease or the DMS footprinting assays CjDnaA was used at 20:1 to 160:1 DnaA:*oriC* ratios while *H. pylori* DnaA was used at up to 80:1 DnaA:*oriC* ratio in P1 (Donczew et al., 2012) and DMS assays (Donczew et al., 2014). We did not observe significant differences in concentration of HisCjDnaA and StrepCjDnaA required for specific unwinding of *C. jejuni oriC*, thus it was concluded that the tags did not interfere with *C. jejuni* DnaA unwinding or DNA binding activities. StrepCjDnaA was preferentially used in analyses that could require a free N-terminus [e.g., long-range interactions in electron microscopy (EM) or putative interactions with Cj1509]. In all analyses, DnaA was supplemented with 3 mM ATP (EM) or 5 mM ATP (footprinting and P1 nuclease assay).

Plasmid pET28Cj1509 was constructed by ligating the PCR-amplified gene using primer pair B4-B5 into BamHI/SalI-digested pET28a(+). Cj1509 protein expression was induced in *E. coli* BL21 by the addition of IPTG to a final concentration of 0.05 mM, followed by an additional incubation for 3 h at 30°C. Cells were centrifuged, resuspended in His-A buffer (50 mM NaH_2_PO_4_, 300 mM NaCl, pH 8.0), sonicated and centrifuged (30 min, 15,000 ×*g*, 4°C). The supernatant was incubated for 1 h at 4°C with HIS-Select Nickel Affinity Gel (Sigma-Aldrich), washed twice with His-A buffer supplemented with 10 mM imidazole and eluted with His-A buffer with increasing concentration of imidazole (20–200 mM).

### DMS Footprinting

*In vitro* DNA modification was performed as described previously (Cassler et al., 1995; Donczew et al., 2014) in a concentration range of 0.4–1.6 μM or 0.5–2.1 μM for 6HisCjDnaA and 6HisCj1509, respectively. Methylated pOC_24 and pOC_IGR4 were used as a template for primer extension (PE) reactions with appropriate primers (see Supplementary Table S2).

### P1 Nuclease Assay

The P1 nuclease assay was conducted as previously described (Donczew et al., 2012). Reaction mixtures contained 300 ng of pOC_IGR plasmid DNA (approximately 10 nM) and 6HisCjDnaA protein (up to 1.6 μM), or a mixture of StrepCjDnaA (up to 0.8 μM) and 6HisCj1509 (up to 1.6 μM), in a total volume of 15 μl. P1 activity was analyzed by SspI restriction enzyme digestion and 1% agarose gel separation or PE analysis. The gels were scanned with a GelDoc Xr+ Imaging System (Bio-Rad).

### Primer Extension (PE) Reactions

The modification sites introduced either by DMS or P1 nuclease were monitored by PE analysis. Reaction conditions, mixture separation and product visualization were conducted as described previously (Jaworski et al., 2016).

### Electrophoretic Mobility Shift Assay (EMSA)

Electrophoretic mobility shift assay was conducted as described previously (Jaworski et al., 2016). The IGR regions were amplified by PCR using IRD800 labeled E1-E2 or FAM-labeled E5-E6 primer pairs (Supplementary Table S2) and specific template pOC_X for IGRX to give IRD800-IGRX or FAM-IGRX (X-respective IGR region, pOC plasmids are described in Supplementary Table S1). The NC control region was amplified by PCR using IRD800 labeled E3–E4 primer pair and specific template pTZ_NC (Supplementary Tables S1, S2) to give IRD800-NC. Probes representing Cj1509 boxes were designed as described previously (Jullien and Herman, 2011). Oligonucleotides (Supplementary Table S2), suspended in H_2_O, were mixed in equimolar concentration (33.3 μM each, in threes: E7–E8–E9 for Cj1509 box 1, E7–E10–E11 Cj1509 box 2 and E7–E12–E13 Cj1509 box 3) and hybridized. 0.4 μM of the complete annealed probe was used in gel shift analyses. IRD800- or FAM-labeled DNA fragments were incubated with 6HisCjDnaA protein (up to 30 nM) or 6HisCj1509 (up to 400 nM) at room temperature for 15 min. Bound complexes were resolved on 4% polyacrylamide gels (1x TBE at 7.5 V/cm) and visualized on an Odyssey CLx Infrared Imaging System (Li-Cor) or Typhoon FLA9500 Variable Mode Imager (GE Healthcare).

### Electron Microscopy (EM)

Electron microscopy was performed as described previously (Spiess and Lurz, 1988; Donczew et al., 2012, 2014) with few modifications. 90 ng (approximately 110 nM) of StrepCjDnaA protein was incubated with 60 ng (approximately 1.4 nM) of pOC_24 plasmid DNA. Complex localization was measured using ImageJ 1.46v software (Schneider et al., 2012). To calculate the binding and distribution of protein, approximately 300 DNA molecules were analyzed.

### *In Silico* Analysis

The prediction of *oriC*-type replication origins in the *C. jejuni* 81116 chromosome was performed using a stepwise procedure, as described previously (Donczew et al., 2012; Jaworski et al., 2016). The DnaA box consensus sequence was generated by WebLogo3 (Schneider and Stephens, 1990; Crooks et al., 2004). The DnaA and Cj1509 box search was performed by Pattern Locator (Mrázek and Xie, 2006). DnaA alignment was prepared by Praline (Bawono and Heringa, 2014).

## Results

We identified the probable *C. jejuni* origin of chromosome replication and characterized its three basic modules: DUE, DnaA boxes and OriBP binding sites in a three-step approach: *in silico* analysis of putative origins, analyses of mini-chromosome replication *in vivo* and DnaA-DNA and Cj1509-DNA interactions *in vitro*.

### *C. jejuni oriC* Is Located Downstream of *dnaA*

DoriC predicted *C. jejuni* 81116 *oriC* for pos. 1324–1482 [ORI92240122] (i.e., *dnaA–dnaN* intergenic region) (Gao et al., 2013). According to this database, the predicted *oriC* contains two DnaA boxes highly similar to *E. coli* perfect DnaA box (no more than one mismatch from 5′-TTATCCACA-3′). However, Epsilonproteobacterial DnaA boxes differ from the perfect *E. coli* DnaA box (Jaworski et al., 2016). Thus, we searched for *E. coli* consensus DnaA box sequence (5′-TTWTNCACA-3′) allowing for two mismatches (Schaper and Messer, 1995). The search also filtered for the presence of a 5′-TCAC-3′ (5–8 nt) sequence to meet more stringent Epsilonproteobacterial criteria (Jaworski et al., 2016). We found a cluster of four DnaA boxes at predicted *oriC* and the putative DUE sequence downstream of the last DnaA box (**Figure 1A** and Supplementary Figure S1). Significantly, a degenerated ‘DnaA trio’ motif (5′-TAG-3′) (Richardson et al., 2016) was found between the DnaA box cluster and the putative DUE. Due to these structural similarities to other *oriC*s of Epsilonproteobacteria, we assumed that replication started at *dnaA–dnaN* intergenic region. However, numerous DnaA boxes were also found in the intergenic regions upstream of *dnaA* (intergenic regions were denoted IGR1–IGR3, *dnaA–dnaN* was denoted IGR4 for clarity of description, **Figure 1** and Supplementary Figure S1). Since the known Epsilonproteobacterial *oriC* regions are bipartite (Jaworski et al., 2016), IGR4-proximate IGR2 or less likely IGR4-distal IGR1, which contain predicted DnaA boxes, may be involved in the initiation of *C. jejuni* replication similarly to Epsilonproteobacterial *oriC1* (Jaworski et al., 2016).

**FIGURE 2:**
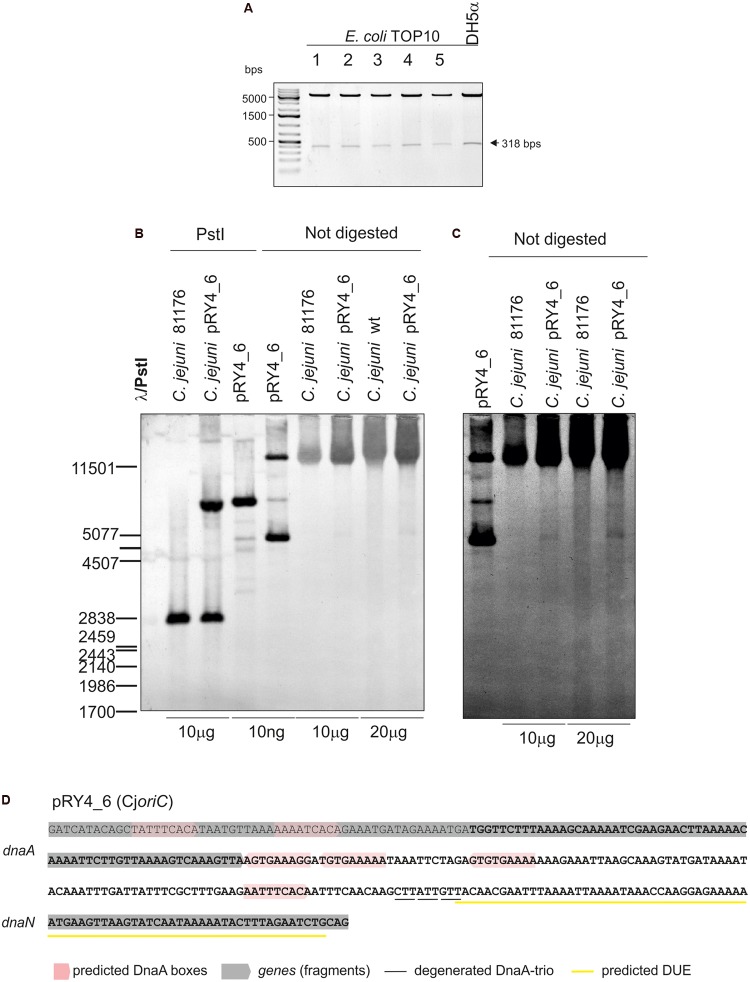
Analysis of pRY4_6 self-replication in *C. jejuni*. **(A)** Gel analysis of plasmids isolated from *E. coli* TOP10 cells transformed with genomic DNA of *C. jejuni* pRY4_6 conjugant strain. Plasmids were digested by EcoRI and PstI. The plasmid propagated in *E. coli* DH5a is shown as a control. **(B)** Southern blot analysis of *C. jejuni* pRY4_6 conjugant strain. *C. jejuni* 81176 wild type and *C. jejuni* pRY4_6 conjugant strains genomic DNA and the pRY4_6 plasmid, isolated from *E. coli*, undigested or digested with PstI, were resolved in 1% agarose gel (Supplementary Figure S3), transferred onto a nylon membrane and probed with the digoxigenin-labeled DNA probe (**Figure 1C**). Southern blot was developed by anti-digoxigenin antibody followed by colorimetric reaction. **(C)** The fragment of the blot, presenting probe hybridisation to not digested DNA, was digitally manipulated to increase intensity of bands corresponding to the self-replicating pRY4_6 and to reduce the background. Adjustment was applied to the whole image. **(D)** Nucleotide sequence of *C. jejuni* DNA, which enabled self-replication of the pRY4_6 minichromosome. The sequence in bold presents DNA region cloned into pRY4, which did not replicate in *C. jejuni*.

Thus, we analyzed the functionality of all four selected IGRs as putative *oriC* (sub-)regions in *C. jejuni.* We used a minichromosome approach, which has been successfully applied to identify or to characterize chromosomal origins of replication of several bacterial species, for example, *E. coli*, *Streptomyces coelicolor*, and *B. subtilis* (Yasuda and Hirota, 1977; Moriya et al., 1992; Zakrzewska-Czerwińska et al., 1995). The minichromosome is a plasmid that contains a chromosomal *oriC* as sequence that supports the plasmid’s autonomous replication in a cell of a given species (Dasgupta and Løbner-Olesen, 2004). In addition, minichromosomes contain selection markers and may contain sequences for propagation of a plasmid (plasmid *oriV*) in a heterologous host strain, which is usually *E. coli*. To study the *C. jejuni oriC*, we used a derivative of a shuttle *E. coli–C. jejuni* plasmid pRY107 as a cloning vector (Yao et al., 1993). We removed Cj*oriV* supporting pRY107 replication in *C. jejuni* and obtained pRY107_d, which was incapable of replicating in *C. jejuni* but contained Ec*oriV* and, thus, could still replicate in *E. coli* (**Figure 1B** and Supplementary Figure S2). pRY107_d was further used for cloning of the IGRs previously determined by *in silico* analyses as putative *C. jejuni oriC* regions (see section “Materials and Methods”). A series of plasmids was obtained containing the intergenic regions IGR1–IGR4 (pRY1, pRY2, pRY3, pRY4, respectively) (**Figure 1B**, Supplementary Table S1 and Supplementary Figure S2). The plasmids were introduced into *C. jejuni* 81–176 via conjugation (see section “Materials and Methods”); pRY107 was introduced in a parallel conjugation and served as a positive control for conjugation, replication and selection in *C. jejuni*. None of the cloned IGR regions supported the replication of the plasmids in *C. jejuni* because no colonies were obtained after conjugation. Conversely, the control pRY107 plasmid replicated in *C. jejuni* because kanamycin-resistant *C. jejuni* conjugant colonies grew on selective plates. The *in silico* analysis indicated that there were numerous DnaA boxes within the *dnaA* gene and other IGR regions, which could be necessary to support IGR4 activity (**Figure 1A**). Therefore, we prepared a series of plasmids, all of which contained IGR4 but differed in the length of DNA extending upstream of IGR4, up to IGR2 (pRY4_6 and data not shown, Supplementary Table S1 and **Figures 1B,C**). Plasmid pRY4_6, which contained IGR4 extended by 120 bp of 3′ of *dnaA* encompassing two additional putative DnaA boxes, was the shortest construct, which, when introduced into *C. jejuni* by conjugation, successfully supported *C. jejuni* growth on selective kanamycin plates. We confirmed by PCR that the genomic DNA isolated from *C. jejuni* pRY4_6 conjugants contained the pRY4_6 plasmid (Supplementary Figure S2B). We also excluded by PCR the possibility that residual *E. coli* DNA contaminated isolated *C. jejuni* genomic DNA (Supplementary Figure S2B). Thus, the pRY4_6 plasmid detected by PCR was carried by *C. jejuni* conjugants. pRY4_6 could be carried by *C. jejuni* as a self-replicating plasmid or could have been integrated into *C. jejuni* chromosome (**Figure 1C**). To confirm that pRY4_6 self-replicated in *C. jejuni*, we isolated pRY4_6 plasmid from *C. jejuni*, transformed *E. coli* with this plasmid, re-isolated it from transformants and digested the extracted plasmid by EcoRI and PstI. The restriction pattern confirmed the authenticity of pRY4_6 (**Figure 2A**). Finally, we performed Southern blot to confirm the presence of self-replicating pRY4_6 plasmid in *C. jejuni*. We resolved undigested and PstI-digested *C. jejuni* genomic DNA and the pRY4_6 plasmid in 1% agarose gel (Supplementary Figure S3) and transferred DNA onto a nylon membrane. The membrane was then incubated with the digoxigenin-labeled, 314 bp-DNA probe, corresponding to the sequence of IGR4 extended by approximately 120 bps of the 3′ *dnaA* sequence (**Figure 1C**). In undigested genomic DNA the probe detected IGR4 within high-molecular weight *C. jejuni* chromosomal DNA, both in *C. jejuni* wild type and *C. jejuni* pRY4_6 conjugant (**Figures 2B,C**). In pRY4_6 conjugant strain, but not in the wild type strain, the additional single band corresponding to intact, self-replicating plasmid DNA was detected. The intensities of bands representing the self-replicating pY4_6 plasmid were low when compared to the signal detected within undigested *C. jejuni* chromosomal DNA, which suggested that only a fraction of cells maintained the self-replicating plasmid, while in majority of *C. jejuni* cells the plasmid recombined with the chromosome. In PstI-digested genomic DNA, in *C. jejuni* wild type strain, the probe detected a single DNA band corresponding to wild type IGR4 genomic locus. In *C. jejuni* pRY4_6 conjugant, the probe detected two bands corresponding to wild type IGR4 genomic locus and pRY4_6 plasmid which integrated into the *C. jejuni* chromosome. Molecular weight and approximately 1:1 stoichiometry of detected bands suggested that recombination occurred via single crossing over (**Figure 1C**). The PCR reaction, performed using F3–F4 primer pair (**Figure 1C**), confirmed the presence of both intact and recombined *oriC* loci in DNA isolated from *C. jejuni* pRY4_6 conjugants (Supplementary Figure S2C), which further confirmed the presence of two populations of *C. jejuni* cells. Altogether, the results showed that *C. jejuni* pRY4_6 is unstable and tends to integrate into the *C. jejuni* chromosome, which is a known and common phenomenon observed for minichromosomes in diverse bacterial species (see section “Discussion”). Nonetheless, since pRY4_6 was re-isolated from *C. jejuni* and was detected as a self-replicating plasmid in southern blot, we conclude, that the a 318 bp fragment containing the intergenic region between *dnaA* and *dnaN* (IGR4) and two predicted DnaA boxes located in the 3′ region of the *dnaA* gene is sufficient to maintain replication of mini-chromosomes. We named this region probable *C. jejuni oriC* (Cj*oriC*) (**Figure 2D**).

### *C. jejuni* DnaA Unwinds Cj*oriC in Vitro*

**FIGURE 3:**
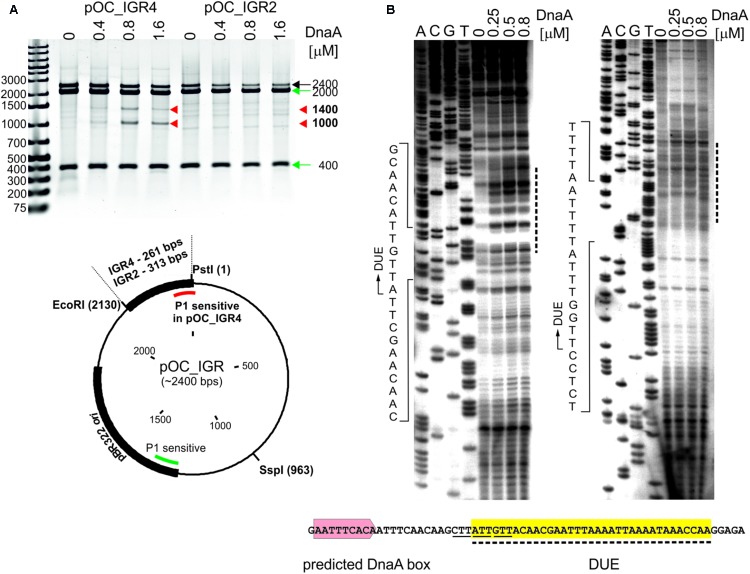
Identification of the DUE in *C. jejuni oriC*. **(A)** Supercoiled plasmids pOC_IGR2 and pOC_IGR4 were incubated with the indicated amounts of 6HisDnaA and successively digested by P1 nuclease and SspI. Digested plasmids were resolved in 1% agarose gel, and the fragments resulting from DnaA-dependent unwinding are indicated by red arrowheads next to the gel, while the DNA fragments resulting from DnaA independent unwinding within the plasmid pBR322 *ori* site are indicated by green arrows. A map of the pOC_IGR plasmids used in the P1 nuclease assay is presented below the gel. The IGR regions, pBR322 plasmid origin of replication and positions of the most important restriction sites are marked. P1-sensitive DnaA-dependent unwinding is distinguished from DnaA-independent by red and green lines, respectively. **(B)** The supercoiled plasmid pOC_IGR4 was incubated with the indicated amounts of 6HisDnaA, digested by P1 nuclease and used as a template for PE reactions with ^32^P-labeled primers C1 and C2. Dashed lines on the right of the PE gel indicate the nucleotides susceptible for the P1 nuclease treatment, while the boundaries of the DUE are marked with continuous-line arrows next to the presented sequences. The sequence of the DUE (dashed line, yellow shadowing), the degenerated DnaA-trio motif (solid line) and predicted DUE-proximal DnaA box (light-red) are presented below the PE gel. Yellow shadowing is used to mark DUE in subsequent figures.

In the next step, we assessed whether the identified Cj*oriC* region is unwound by DnaA *in vitro* since the presence of the region that undergoes specific DnaA-dependent unwinding is the most unequivocal *in vitro* indication of *oriC* functionality. To experimentally validate DNA unwinding and to identify the DnaA-dependent DUE position in predicted Cj*oriC*, P1 nuclease assay was applied (Sekimizu et al., 1987). A plasmid containing the IGR4 region was constructed (pOC_IGR4), and the *C. jejuni* DnaA protein was purified (see section “Materials and Methods,” **Figure 3A**, Supplementary Table S1 and Supplementary Figure S4). As a control, we constructed a pOC_IGR2 plasmid containing IGR2 (Supplementary Table S1 and **Figure 3A**), which was predicted to exhibit significant helical instability (data not shown), however, it was unable to support minichromosomal replication in *C. jejuni* (**Figure 1B**). The supercoiled plasmids were incubated with increasing amounts of DnaA protein and digested with P1 nuclease to cleave the resulting single-stranded DNA regions. Subsequently, site-specific digestion by SspI excised the DNA fragment from the plasmid. The size of the fragment allowed us to estimate the position of a region unwound by DnaA. In the case of pOC_IGR4, DNA fragments of approximately 1000 and 1400 bp were excised by P1/SspI, indicating specific formation of a single-stranded DNA within the *CjoriC* region (**Figure 3A**). pOC_IGR2 was only linearized by SspI. Thus, DnaA-dependent unwinding occurred in IGR4, but not in IGR2 (**Figure 3A**). IGR4 represents Cj*oriC* lacking the two DnaA boxes distal to DUE (**Figure 2D**), but it was previously shown that not all DnaA boxes are required for DUE unwinding in the *oriC* cloned into a plasmid (Ozaki and Katayama, 2012; Donczew et al., 2014; Sakiyama et al., 2017). Regardless of the DnaA presence or concentration, all lanes contained additional DNA fragments of 400 and 2000 bp because the plasmids were also unwound at a site corresponding to the plasmid origin of replication (Kowalski et al., 1988). In addition, a fraction of pOC_IGR4 molecules was unwound simultaneously at IGR4 and plasmid origin, which resulted in three DNA fragments: one 400 bp and two 1000 bp. Therefore, the overall intensity of the 1000 bps DNA band on the gel was higher than that of 1400 bp band (**Figure 3A**).

To precisely determine the unwound regions, primer extension (PE) reactions with ^32^P-labeled primers were performed using the P1-digested pOC_IGR4 plasmid template (**Figure 3B**; the primers are specified in Supplementary Table S2). The primers hybridized to the template DNA approximately 40–80 bp away from the *in silico*-predicted DUE region within IGR4, which was extended by Taq polymerase until the P1 nuclease digestion site was encountered. The detailed PE analysis confirmed that the IGR4 region underwent DnaA-dependent unwinding, and thus it contained the DUE sequence (**Figure 3B**). The DUE encompasses approximately 35 bps and contains 19% GC residues (overall chromosomal GC content is 30.5%). The main part of the identified DUE region is an AT-rich region, which is a typical feature of bacterial origins. Analysis of the DUE sequence did not reveal any repeats similar to 13-mer *E. coli* L, M, R repeats. We detected a degenerate DnaA-trio (5′-TAG-3′), but no GC-rich region was found in the *C. jejuni oriC* region (Richardson et al., 2016).

Taken together, the above *in vivo* (minichromosomes) and *in vitro* (P1 plasmid unwinding) data indicate that IGR4 extended by approximately 120 bps of a 3′ region of *dnaA* is probably the functional *C. jejuni oriC.*

### DnaA Boxes Are Present in *oriC* and in the *oriC*-Vicinal Region

The DNA is unwound by the DnaA protein bound to DnaA boxes at *oriC*. The *in silico* analysis predicted numerous DnaA binding sites in the vicinity of Cj*oriC* (**Figure 1A** and Supplementary Figure S1). Accordingly, the gel shift results indicated that IRD800-labeled DNA fragments comprising IGR1, IGR2, and IGR4, for which the DnaA boxes were predicted, were bound by DnaA, while no binding was observed for a fragment comprising IGR3, which contained only 1 predicted, apparently non-optimal DnaA box sequence (Supplementary Figure S5). The number of distinct nucleoprotein complexes that formed between DnaA and IRD800-IGR4 was higher than between DnaA and IRD800-IGR2 or IRD800-IGR1, indicating a higher number of DnaA binding sites at IRD800-IGR4 than at IRD800-IGR2 and IRD800-IGR1.

**FIGURE 4:**
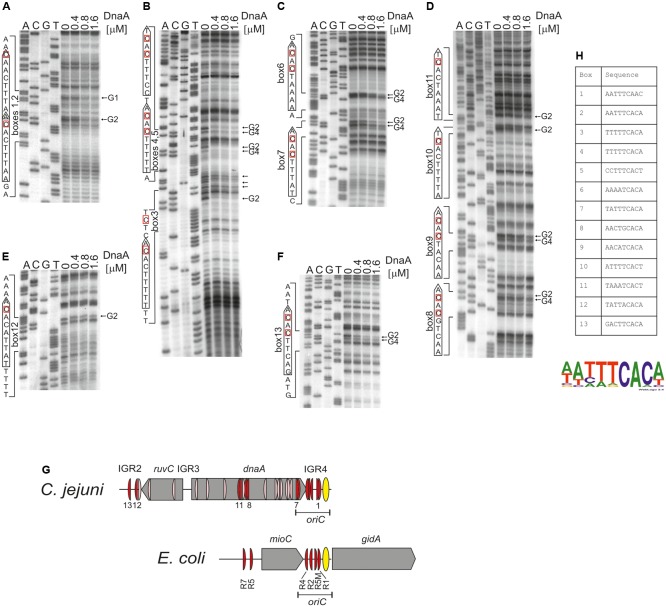
Identification of DnaA boxes in the *C. jejuni* IGR2, IGR4 regions and the *dnaA* gene by *in vitro* DMS footprinting. **(A–F)** pOC_24 was incubated with the indicated concentrations of the 6HisDnaA protein, methylated with DMS and used as a template for PE reactions; primers used to map DnaA boxes are specified in Supplementary Table S2. Protected guanosine residues (G) are indicated by arrows, and the complementary cytosine residues in the DnaA box are indicated by red boxes. Densitometric plots are presented in Supplementary Figure S6. **(G)** Schematic presentation of the localization of DnaA boxes identified *in silico* (light red) and detected by DMS footprinting (red). The *E. coli oriC* region is presented for comparison of the most characteristic *oriC* features; only strong R-type DnaA boxes are marked (Hansen et al., 2007; Leonard and Grimwade, 2015). **(H)** Sequences of *in vitro*-identified *C. jejuni* DnaA boxes. The WebLogo was used to create a consensus sequence of the *C. jejuni* DnaA box.

Therefore, the next step was to precisely identify DnaA boxes at *C. jejuni oriC* by dimethyl sulfate (DMS) footprinting (Sasse-Dwight and Gralla, 1991; Cassler et al., 1995; Shaw and Stewart, 2009) and to establish the *C. jejuni* DnaA box consensus sequence. Briefly, DMS methylates guanine or adenine residues. DMS footprinting allows the detection of DNA sequences that are bound by a protein and thus protected by the protein against guanine or adenine methylation. These protected residues are distinguishable as DNA bands with a decreased intensity on a footprinting gel. The pOC_24 plasmid (Supplementary Table S1) containing the IGR2*-ruvC-*IGR3*-dnaA*-IGR4 region was incubated with increasing concentrations of the *C. jejuni* DnaA protein and methylated by DMS. To determine protein binding sites, sets of primers (Supplementary Table S2), that were complementary to the upstream regions of predicted DnaA boxes were used in PE reactions. We detected multiple G residues that were protected by DnaA in IGR4 (**Figures 4A–C** and Supplementary Figure S6). The subsequent comparison of the DNA sequences in the vicinity of protected G residues allowed to classify the identified DnaA binding sites as DnaA boxes, because they resembled DnaA boxes characterized in other bacterial species (Wolański et al., 2014) (Supplementary Figure S7) (see also section “Discussion”). In total we identified five DnaA boxes in the IGR4 intergenic region and two DnaA boxes enclosed within the 3′ end of the *dnaA* gene that were essential to support minichromosome replication in *C. jejuni* as discussed above (**Figure 2D**). Thus, the *C. jejuni oriC* contained seven DnaA boxes bound by DnaA *in vitro*, from which one DnaA box (5′-AATTTCAAC-3′, DnaA box 1) was not predicted *in silico*, because of absence of the Epsilonproteobacterial core sequence (Supplementary Figure S6B). The *oriC* region preserved the general features of a typical bacterial origin of replication, namely, the distance (approximately 1–2 helical turns) and spatial orientation between the DUE and CjDnaA box 1 (**Figure 4G**) and the fact that CjDnaA box 1 is accompanied by a second DnaA box 2 in head-to-tail orientation. This array has been proposed to be essential for the formation of a functional orisome and DUE unwinding in a few bacterial species including *H. pylori* (Donczew et al., 2014). The unique feature of *C. jejuni oriC* is the possible requirement for DnaA boxes located in the *dnaA* gene for the activity of *oriC in vivo* (see section “Discussion”).

We additionally analyzed DnaA binding to two other regions outside *oriC* at which DnaA boxes were predicted: IGR2, which is supposed to contain two DnaA boxes and the middle region of *dnaA* in which four DnaA boxes were predicted (Supplementary Figures S1, S6B). The DMS footprinting analysis confirmed DnaA binding to four of those predicted DnaA boxes (DnaA binding sites 9–11 and 13), while it allowed to identify two new DnaA binding sites: 8 (5′-AACTGCACA-3′) and 12 (5′-TATTACACA-3′), which were not predicted due to deviation from the core of the typical Epsilonproteobacterial DnaA box (**Figures 4D–F** and Supplementary Figure S6). Please note, that our analysis do not preclude further binding of DnaA to other oppositely oriented or non-clustered DnaA boxes, not detected by using by using a single PE primer. Nonetheless DMS footprinting and *in silico* analyses indicated a high number of DnaA binding sites in the *oriC*-proximal region (**Figure 4G**); however, they were more scattered in regions outside of *oriC* than those enclosed within *C. jejuni oriC*, with the exception of DnaA boxes in the middle and in the 3′ region of *dnaA* (see also below). The sequences of 13 *in vitro*-determined DnaA binding sites were assembled to generate a logo of the *C. jejuni* DnaA box sequence, 5′-NHHWDCAMH-3′ (Supplementary Figure S7), with the majority of boxes at *oriC* following the more stringent Epsilonproteobacterial DnaA box core consensus 5′-WWHTTCACW-3′ sequence (**Figure 4H** and Supplementary Figure S7) (see also section “Discussion”).

### The DnaA-*oriC* Nucleoprotein Complex Engages *oriC*-Proximal DNA

**FIGURE 5:**
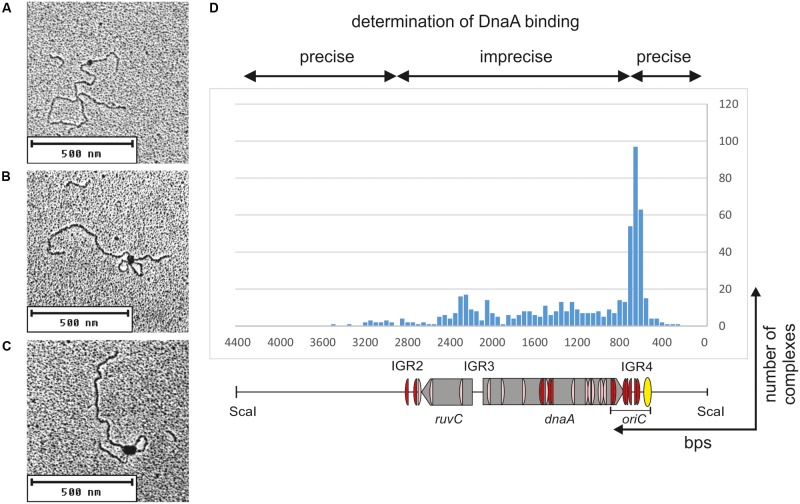
Electron microscopy analysis of the StrepCjDnaA interaction with pOC_24 plasmid. The plasmid was incubated with StrepCjDnaA protein, fixed with glutaraldehyde and digested with ScaI. Representative pictures present the following: **(A)** StrepCjDnaA binding to the IGR4 region. **(B)** The simultaneous interaction of StrepCjDnaA with IGR4 and DnaA boxes in the *dnaA* gene. **(C)** Large StrepCjDnaA complex interactions with multiple sites along IGR4-*dnaA.* **(D)** Histogram of complexes of supercoiled pOC_24 with StrepCjDnaA. The most characteristic features of the plasmid are shown below the histogram. The distribution of the complexes was calculated based on an analysis of 300 molecules.

*C. jejuni oriC* is most likely monopartite. However, the results presented thus far indicated the presence of numerous DnaA binding sites in *oriC*-vicinal regions, such as *ruvC, flgE*, and *dnaA* genes or the IGR1, IGR2, and IGR4 intergenic regions (**Figures 1A**, **4G**). The binding of DnaA to IGR1 and IGR2 intergenic regions was confirmed by gel shift and footprinting assays (Supplementary Figure S5 and **Figure 4**). The results suggested that the nucleoprotein complex formed by *C. jejuni* DnaA might extend beyond the Cj*oriC* region required to support replication of the minichromosome (pRY4_6, **Figure 1B**). Such auxiliary binding sites may play regulatory roles in the initiation of *C. jejuni* chromosome replication or indicate further functions of DnaA, for example, in chromosome maintenance or structure. Thus, to better characterize DnaA binding to *oriC* proximal regions and, especially, to monitor intermolecular interactions between DnaA, we further analyzed the binding of DnaA to a plasmid that contained the entire region between *flgE* and *dnaN* by electron microscopy (EM) (**Figure 5**). The supercoiled pOC_24 plasmid that contained IGR2-*ruvC-*IGR3*-dnaA*-IGR4 (Supplementary Table S1) was incubated with the StrepCjDnaA protein. The StrepCjDnaA protein contained a C-terminal Strep-tag and a native N-terminus, which might be essential for long-range protein–protein interactions (Zawilak-Pawlik et al., 2017). The nucleoprotein complexes were subsequently stabilized by glutaraldehyde crosslinking and digested by ScaI to linearize the plasmid molecules. EM analysis revealed that the majority (94%) of the analyzed plasmid molecules were bound by DnaA (**Figure 5**). Two predominant kinds of nucleoprotein complexes were formed: (i) plasmid molecules with a single protein complex bound to a single plasmid region (type 1 complexes, **Figure 5A**) and (ii) looped DNA structures with a relatively large protein complex (or complexes) bound to at least two separated DNA regions (type 2 complexes, **Figures 5B,C**).

The type 1 complexes constituted 37% of all bound molecules. The distance measurements confirmed the binding of DnaA to the *oriC* region in 52% of the single nucleoprotein complexes (19% of all complexes), while 15% of the type 1 protein complexes were formed by DnaA binding between *oriC* and IGR2 (5.6% of all complexes, **Figure 5D**). Additionally, 33% of the type 1 complexes (12% of all complexes) were formed outside of *C. jejuni* DNA (i.e., on a DNA of the vector pOC), and thus they should be considered non-specific.

The type 2 complexes constituted 63% of all bound molecules. The distance measurements between the plasmid ends and the protein core confirmed the simultaneous binding of DnaA to *oriC* and to the region located between IGR2 and *oriC* in 66% of the complexes (42% of all bound plasmid molecules, **Figures 5B,C**). However, in the type 2 complexes, DnaA bound to and condensed a large portion of *ruvC-*IGR3*-dnaA-oriC* DNA, with only a short unbound DNA stretch being looped out into 1 or 2 short loops. Thus, only the borders of DnaA binding (i.e., the length of the plasmid between ScaI-IGR2 and *oriC*-ScaI) could be relatively precisely measured along a plasmid molecule. Approximately 30% of the type 2 complexes (19% of all complexes) were bound at IGR2-*ruvC-*IGR3*-dnaA* without binding to *oriC*, and 4% of the type 2 complexes (2% of all complexes) were formed in non-specific regions (**Figure 5D**).

Taken together, the EM analysis indicated that in two types of complexes, type 1 and type 2, DnaA exhibited a higher affinity or more stable binding toward *oriC* than toward other regions because 60% of the complexes involved *oriC* (**Figure 5D**). Nonetheless, the majority of the complexes also included other regions of *C. jejuni* DNA (36 and 63% of types 1 and 2 complexes, respectively). This result indicated that (i) *C. jejuni* DnaA bound to multiple sites along DNA and (ii) DnaA exhibited high dimerization or oligomerization potential, and probably the ability to establish long-range interactions. These two findings suggest DnaA activity in processes beyond initiation of *C. jejuni* chromosome replication (see section “Discussion”).

### The Cj1509 Orphan Response Regulator Interacts With *CjoriC* and Inhibits DUE Unwinding

The orphan response regulator HP1021 was recently identified in *H. pylori* as a negative replication initiation regulator (Donczew et al., 2015). Since the two bacteria are related and the HP1021 protein is conserved and unique in Epsilonproteobacteria, we decided to analyze the role of the *C. jejuni* homolog Cj1509 in the initiation of *C. jejuni* chromosome replication.

**FIGURE 6:**
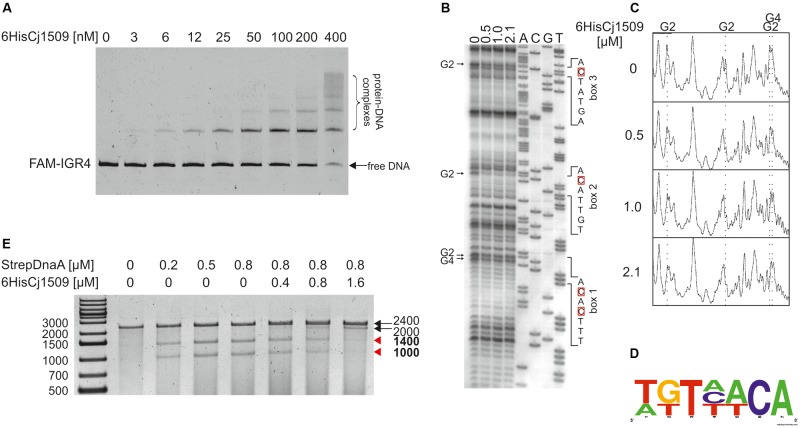
Analysis of 6HisCj1509 protein binding to the IGR4 region and influence of 6His1509 on DUE unwinding. **(A)** EMSA analysis of nucleoprotein complex formation by 6His1509 and IGR4. FAM-labeled IGR4 (310 bps) was incubated with the indicated amounts of 6HisCj1509 protein. The reaction mixtures were separated by electrophoresis on a 4% polyacrylamide gel. **(B)** Identification of 6HisCj1509 binding sites by DMS footprinting. pOC_IGR4 was incubated with the indicated concentrations of the 6HisCj1509 protein, methylated with DMS and used as a template for PE reactions with the ^32^P-labeled C1 primer complementary to the lower strand. Protected guanosine residues (G) are indicated by arrows presented next to the gel image, while complementary cytosine residues (C) of the 6HisCj1509 binding sequence are denoted by red boxes. **(C)** Densitometric plot of the DMS analysis; vertical, dotted lines and symbols above the plot correspond to the protected G residues. **(D)** The consensus sequence bound by *C. jejuni* 1509 generated with WebLogo based on the identified 6HisCj1509 binding sites. **(E)** The influence of 6HisCj1509 on DUE unwinding by DnaA. The pOC_IGR4 plasmid was incubated with the indicated amounts of 6HisCj1509 and StrepCjDnaA proteins, treated with P1 nuclease and restriction digested with SspI. DNA fragments were analyzed by separation on a 1% agarose gel and ethidium bromide staining. A map of the pOC_IGR4 plasmid is presented in **Figure 2**. Fragments of DnaA-specific unwinding are indicated by red arrowheads next to the gel.

To determine the affinity of Cj1509 for DNA, a gel-shift assay was conducted. For this purpose, a recombinant 6HisCj1509 protein was purified (see section “Materials and Methods,” Supplementary Figure S4). A PCR-amplified *CjoriC* region was incubated with purified 6HisCj1509, and the complexes were resolved in a polyacrylamide gel (**Figure 6A**). The Cj1509 protein bound DNA *in vitro* and three nucleoprotein complexes were formed, suggesting either multiple Cj1509 binding sites or cooperative binding of Cj1509 with *oriC*. Therefore, we decided to precisely determine the sequences bound by Cj1509 at *oriC* by DMS footprinting. The pOC_IGR4 plasmid (Supplementary Table S1) was incubated with increasing amounts of 6HisCj1509 and methylated by DMS. Nucleotides protected by the Cj1509 protein were detected by subsequent PE using the C1 primer (Supplementary Table S2) complementary to the upstream region of the DUE (**Figure 6B**). The densitometric analysis revealed four G residues that were specifically protected by Cj1509 already at the lowest tested Cj1509 concentration (0,5 μM, **Figure 6C**); further increase in Cj1509 concentration did not greatly increase the protection. The DNA sequences in the vicinity of protected residues were assembled to generate a consensus sequence of the Cj1509 box 5′-WKTHWCA-3′ (**Figure 6D**), which is less stringent than that of the HP1021 box (5′-TGTTWCW-3′). To confirm binding of Cj1509 to each of the identified boxes gel shift assay was performed. Cj1509 was incubated with FAM-labeled probes representing each of the Cj1509 boxes and the complexes were resolved in a polyacrylamide gel (Supplementary Figure S8). The lower intensity of the complexes formed by Cj1509 with boxes 1 and 3 than that formed with the box 2 suggested that Cj1509 exhibits higher affinity toward the Cj1509 box 2 than toward boxes 1 or 3. Notably, the Cj1509 box 2 (5′-TGTTACA-3′) has the highest similarity (no mismatches) to the HP1021 box consensus sequence compared to Cj1509 box 1 (5′-TTTCACA-3′) or Cj1509 box 3 (5′-AGTATCA-3′) (each of which has two mismatches).

**FIGURE 7:**
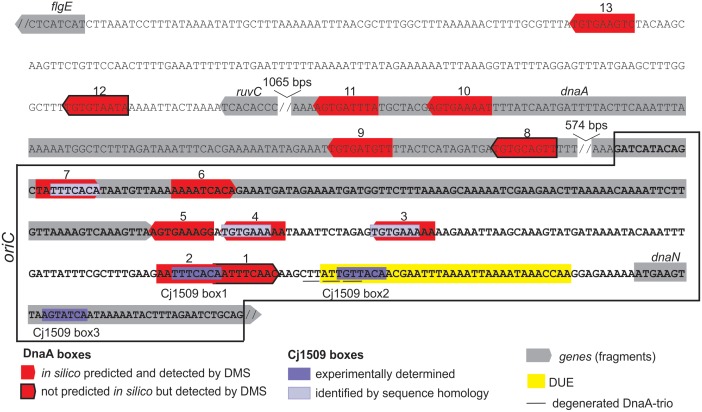
Summary of the most characteristic features identified by DMS, P1 nuclease and minichromosome maintenance assay in *C. jejuni oriC*. The minimal sequence sufficient to support autonomous replication of the minichromosome is boxed. The discontinuity of the DNA sequence is marked by a double slash.

Two out of three Cj1509 boxes were located within sequences important for *oriC* activity: DnaA box 2 and DUE (**Figure 7**). The DnaA box 2 is probably important for assembly of a DnaA oligomer capable of DNA unwinding. Thus, overlapping DnaA and Cj1509 interaction sites suggested that these two proteins compete for binding to DNA. The 3′ DUE sequence is probably crucial for the formation of a complex between the DnaA filament and ssDNA. To analyze the influence of Cj1509 on DnaA-dependent DNA unwinding, we performed a P1 nuclease test. DnaA was incubated with pOC_4 plasmid, which led to significant unwinding at the DUE (**Figure 6E**). The addition of 6HisCj1509 at an equimolar or higher concentration than DnaA inhibited DUE unwinding, which indicated that the binding of Cj1509 to *C. jejuni oriC* inhibited DnaA-dependent DNA unwinding, as previously shown for HP1021 in *H. pylori*.

## Discussion

Correct timing and synchronization of chromosome replication with other processes are vital for bacterial fitness. Bacterial *oriCs* have been proposed to act as centralized information processors that receive and transmit information reflecting the current state of the bacterial cell (Marczynski et al., 2015). Thus, the identification of *oriC* and characterization of the mechanism of initiation of chromosome replication are crucial for studies on the bacterial cell cycle, as well as adaptation to the environment and host-pathogen interactions. In this work, we present the first data pinpointing the *oriC* of the *C. jejuni* chromosome and identifying possible regulators of replication initiation in this bacterium.

### Three Modules of *C. jejuni oriC*

We found that *C. jejuni oriC* is composed of the three standard replication origin modules: DnaA-box cluster, DUE and OriBP binding site, all of which were located in the *dnaA* – *dnaN* locus (**Figure 7**). This localization of *C. jejuni oriC* is typical for many bacteria. Unlike other known Epsilonproteobacterial origins, which are most likely bipartite, we showed herein that *C. jejuni oriC* is probably monopartite because the single intergenic region between *dnaA* and *dnaN*, accompanied by 2 DnaA boxes at the 3′ end of *dnaA*, was sufficient to sustain self-replication of a minichromosome (see also below). In addition, the looped DNA structures typical for bipartite origins (Krause et al., 1997; Donczew et al., 2012; Jaworski et al., 2016) were not observed in EM upon *C. jejuni* orisome formation. The reason for the presence of a mono- or bipartite origin is unknown. It has been proposed that although the *E. coli* DnaA oligomer is assembled on a monopartite origin, it is structurally divided into sub-oligomers of different functions (DNA opening, DnaB loading) (Rozgaja et al., 2011; Ozaki et al., 2012; Shimizu et al., 2016). Thus, in bipartite origins, the DnaA sub-oligomers, which are additionally spatially divided, may provide another level of control during orisome formation.

There are at least 7 DnaA boxes within *C. jejuni oriC* that are bound by DnaA *in vitro* (see also Discussion below). They do not form any clear-cut oligomerization pattern, similar to oppositely oriented R1-I2 and C3-R4 DnaA box arrays in *E. coli* (Rozgaja et al., 2011; Ozaki et al., 2012; Shimizu et al., 2016). Rather, the boxes are grouped in three divergently oriented sub-arrays (DnaA boxes 1–2, 3–4–5, and 7–8), providing a scaffold for a DnaA oligomer of unknown composition and structure. Bacterial origins identified to date are highly variable in number, spacing and orientation of DnaA boxes, and there is still no general understanding of the DnaA filament assembly with respect to the array of DnaA boxes. Interestingly, DnaA boxes 6–7, which are located within the *dnaA* gene, were shown to sustain replication of *C. jejuni oriC* as minichromosome, while the cluster of five DnaA boxes of the intergenic region IGR4 was not. It has been shown previously that in *Bdellovibrio bacteriovorus*, in which *oriC* is also located in the *dnaA-dnaN* intergenic region, the two DnaA boxes in the 3′ region of *dnaA* are bound by DnaA *in vitro*; however, it is not known whether these boxes are required for the initiation of *B. bacteriovorus* chromosome replication (Makowski et al., 2016). It should be noted that DnaA boxes required to support *oriC* activity on a minichromosome or on a chromosome might be different (Bates et al., 1995; Weigel et al., 2001; Riber et al., 2009). Studies on *E. coli oriC* have shown that mutations of some boxes, including the DUE-distal R4 DnaA box corresponding to DUE distal *C. jejuni* DnaA boxes 6–7 (**Figures 4**, **7**), are not tolerated on minichromosomes while they can be mutagenized on chromosomal *oriC.* However, such mutations, although tolerated, often trigger perturbations in the initiation of chromosome replication and bacterial fitness, and thus they are conditionally required (Bates et al., 1995; Stepankiw et al., 2009). Moreover, minichromosomes are often kept at low copy number, they are unstable and tend to integrate into chromosomes (Zakrzewska-Czerwińska and Schrempf, 1992; Duret et al., 1999; Weigel et al., 2001; Cordova et al., 2002; Lartigue et al., 2003). *E. coli* minichromosomes are exceptional when compared to minichromosomes of other species because they self-replicate at relatively high-copy number [(Løbner-Olesen et al., 1987; Løbner-Olesen and von Freiesleben, 1996) and references herein]. Thus further studies and detailed mutagenesis of DnaA boxes directly within *C. jejuni* chromosomal *oriC* are required to analyze the role and essentiality of DnaA binding sites for *C. jejuni oriC* activity upon initiation of chromosome replication *in vivo*.

Five DnaA boxes at *C. jejuni oriC* resemble the typical Epsilonproteobacterial DnaA box consensus sequence, in which a core sequence 5′-TTCAC-3′ (4–8 nt) is strictly conserved; the DnaA box 1 differs at the 8th position (C→A), while the DnaA box 6 differs at the 4th position (T→A). However, *C. jejuni* DnaA, unlike other Epsilonproteobacterial DnaAs, also recognizes DnaA boxes in which the thymine residue at the 5th position is substituted by another nucleotide (guanine in DnaA box 8 or adenine in DnaA box 12) (see also below).

The DUE (ca. 35 bps, 19% GC) is preceded by twin DnaA boxes 1–2, which overlap by 1 bp. The boxes are followed by ca. 5 bps that probably remains double-stranded upon DUE unwinding. The GC-rich sequence between DnaA boxes and the DUE, which occurs in some origins (Richardson et al., 2016), was not present in *C. jejuni*. Similarly, the DnaA trio consensus motif, which was previously reported to be important for ssDNA binding by DnaA upon DNA unwinding (Richardson et al., 2016), was detected in *C. jejuni oriC*, however, its sequence was degenerated when compared to the consensus 5′-TAG-3′ sequence. It should be noted that the DnaA trio motif is very short, and it is difficult to predict the mismatches that preclude its activity. In addition, it is not known why these motifs are conserved in many but not in all bacterial origins and how ssDNA is bound by bacteria that lack the obvious DnaA-trio motif (e.g., *E. coli*).

We also identified OriBP binding sites, i.e., sequences that are bound by the orphan response regulator Cj1509. There are two experimentally determined Cj1509 binding sites at Cj*oriC*, Cj1509 boxes 1 and 2; the Cj1509 box 3 is located within the *dnaN* gene (**Figure 7**). However, the sequence of Cj1509 box 1 (5′-TTTCACA-3′) was found at three other locations in *oriC* (**Figure 7**). The position of Cj1509 boxes did not seem to be distributed randomly because they overlap with the DnaA boxes (2 and possibly 3, 4, and 7) and the DUE. By competition with the binding sites, Cj1509 potentially interfered with DnaA-*oriC* interactions and precluded DNA unwinding (**Figure 6B**). A similar mechanism was observed in *H. pylori*, in which HP1021, a homolog of Cj1509, bound to DnaA boxes and the DUE, leading to the inhibition of DUE unwinding. Thus, HP1021 has been proposed to act as a repressor of chromosome replication (Donczew et al., 2015). A similar mechanism of inhibition has been previously proposed for *E. coli* and *M. tuberculosis.* It is postulated that under anaerobic conditions, *E. coli* ArcA∼P binds to the AT-rich region of *oriC* and inhibits DNA unwinding (Hwang and Kornberg, 1992b; Lee et al., 2001). *M. tuberculosis* MtrA∼P binds to *oriC* and also the promoter of *dnaA* and inhibits the initiation of *M. tuberculosis* chromosome replication (Fol et al., 2006; Purushotham et al., 2015). The signal that triggers MtrA phosphorylation is still unknown, but it is linked to the infection of macrophages (Fol et al., 2006). Thus, *E. coli* and *M. tuberculosis* proteins control chromosome replication and cell cycle progression in response to environmental conditions, including the stage of host infection. By analogy, Cj1509 and HP1021 might be activated by external stimuli, and these factors might regulate the initiation of chromosome replication in response to host–pathogen interactions. No changes in the level of Cj1509 expression were observed upon infection (de Vries et al., 2017). However, it is likely that, similarly to HP1021 (Müller et al., 2007), post-translational modification of Cj1509 rather than a change in expression level is important for Cj1509 function. Therefore, further studies are required to identify the mechanism of Cj1509 activation and signal transduction.

### The Roles of DnaA Boxes and Cj1509 Binding Sequences Beyond the Initiation of Chromosome Replication

DnaA boxes are also found outside *oriC*, which suggests that DnaA plays a role in processes other than initiation complex formation and DNA unwinding. The *C. jejuni* 5′-NHHWDCAMH-3′ DnaA box consensus sequence derived from 13 experimentally determined DnaA binding sites is relatively relaxed when compared to other Epsilonproteobacterial or *E. coli* DnaA boxes (Jaworski et al., 2016), including the 5th position of the DnaA box previously shown to be strictly conserved in Epsilonproteobacteria (Jaworski et al., 2016). Hence, *C. jejuni* DnaA is exceptional among the Epsilonproteobacteria studied to date. However, the *C. jejuni* DnaA amino acid sequence of the DNA binding helix-turn-helix motif within domain IV is highly similar to that of other Epsilonproteobacteria (Supplementary Figure S9) (Fujikawa et al., 2003; Tsodikov and Biswas, 2011; Jaworski et al., 2016). It should be noted that our studies did not allow to distinguish between low- and high-affinity DnaA binding sites, which differ in nucleotide sequence. For example, *E. coli* low affinity I-DnaA binding sites usually differ by 3–4 bases from high affinity R-type DnaA boxes (McGarry et al., 2004) while 6-mer τ DnaA binding sites are shorter than 9-mer R-type DnaA boxes (Kawakami et al., 2005). Thus, herein determined *C. jejuni* DnaA box consensus sequence may be relaxed because it includes high- and low- affinity DnaA binding sites. On the other hand, *B. bacteriovorus* DnaA box sequence was shown to be conserved within seven nucleotides, while the two nucleotides at the 5′ sequence of DnaA box were not conserved (Makowski et al., 2016). Therefore it is possible that some bacterial DnaAs specifically recognize sequences shorter than typical 9-mer DnaA box sequences. Thus, further studies are required to explain the loose *C. jejuni* DnaA specificity for DnaA consensus sequences.

Twenty DnaA boxes were predicted *in silico* in the vicinity of *C. jejuni oriC* (IGR2-*ruvC*-IGR3-*dnaA*). The binding of DnaA to the *oriC* proximal region was also observed by electron microscopy, and we confirmed the binding of CjDnaA to four randomly chosen DnaA boxes by DMS footprinting (DnaA boxes 9–11 in *dnaA* and 13 in IGR2); additionally we identified 2 DnaA boxes that were not predicted (DnaA box 8 in *dnaA* and 12 in IGR2) (Supplementary Figure S6). DnaA boxes 12 and 13 were separated by 104 bps, which likely excluded cooperativity in DnaA-DNA interactions and indicated that *C. jejuni* DnaA could efficiently recognize single DnaA boxes. There are numerous putative DnaA boxes on the *C. jejuni* 81116 chromosome. For example, there are 2659 DnaA boxes that follow the stringent *C. jejuni* DnaA box consensus sequence 5′-WWHWTCACW-3′ (Mrázek and Xie, 2006). Excluding the *oriC* region, the DnaA boxes were evenly distributed along the chromosome. The role of DnaA boxes scattered along the chromosome is not known. As observed by EM, DnaA bound to *oriC* and proximal DnaA boxes formed a large nucleoprotein complex that might affect the activity of the initiation complex. Thus, the *oriC* proximal DnaA boxes (**Figures 4**, **5**) might play a regulatory function in the control of *C. jejuni* chromosome replication. The genome-wide scattered boxes located in the intergenic regions (100 DnaA boxes, 3.7% of all the boxes) might contribute to regulation of gene expression, similarly to *E. coli* or *B. subtilis* (Messer and Weigel, 2003; Washington et al., 2017). *C. jejuni* DnaA was shown to organize DNA into higher order structures such as loops and wraps (**Figure 5**). Thus, we speculate that DnaA participates in the global control of the nucleoid structure, especially since *C. jejuni* chromosome lacks many nucleoid associated proteins such as H-NS, IHF, and Fis (Shortt et al., 2016), which help to maintain the chromosome structure in other bacteria (Browning et al., 2010; Badrinarayanan et al., 2015; Dame and Tark-Dame, 2016). However, further experimental studies are required to determine the actual DnaA binding sites in the *C. jejuni* chromosome, especially since *in silico* predictions of DnaA boxes often overestimate the number of actual DnaA binding sites. Moreover, binding of DnaA to DNA might differ *in vivo* and *in vitro* (Smith and Grossman, 2015).

The actual binding sites of Cj1509 on the *C. jejuni* chromosome are difficult to predict because the Cj1509 consensus 5′-WKTWWCA-3′ sequence is too relaxed to provide reliable data for *in silico* analysis. Preliminary results suggested that Cj1509 exhibits the highest affinity toward Cj1509 box 2 (5′-TGTTACA-3′). However, further studies are required to better characterize Cj1509 affinity to individual Cj1509 boxes and, since there are multiple Cj1509 binding sites at *C. jejuni oriC*, to determine whether Cj1509-DNA interactions might exhibit cooperativity. Nonetheless, Cj1509 could control the expression of selected *C. jejuni* genes in response to unknown stimuli; however, Cj1509 has been reported to be non-essential in some strains (de Vries et al., 2017). Moreover, in different laboratories, Cj1509 has been shown to be an essential and non-essential gene in the same strain, e.g., *C. jejuni* 11168 (Metris et al., 2011; de Vries et al., 2017; Mandal et al., 2017). Thus, it is possible that strain-specific genome content and growth conditions determine the actual role of Cj1509 in *C. jejuni* physiology.

In summary, we identified and characterized the probable *oriC* region of *C. jejuni*. We anticipate that these studies will initiate further research on the structure and dynamics of the *C. jejuni* chromosome, which in turn will facilitate studies on the *C. jejuni* life cycle in the context of its biology and pathogenicity. The results are also important for further comparative investigations of the initiation of chromosome replication and other cellular processes throughout the whole class of Epsilonproteobacteria, which include established and emerging pathogens associated with gastrointestinal diseases and/or reproductive disorders in animals, as well as non-pathogenic symbiotic or free-living species (Eppinger et al., 2004; Gupta, 2006; Nakagawa and Takaki, 2009; Waite et al., 2017). Such diverse life styles of Epsilonproteobacteria might be reflected by the diversity of the initiation or regulatory factors involved in the initiation of chromosome replication of species inhabiting various ecological niches.

## Author Contributions

AZ-P, PJ, RD, and KS conceived and designed the experiments. AZ-P and PJ performed the experiments. CW prepared *in silico* data. TM provided EM facility. PJ and AZ-P analyzed the data and wrote the paper.

## Conflict of Interest Statement

The authors declare that the research was conducted in the absence of any commercial or financial relationships that could be construed as a potential conflict of interest.
