# Supplementary material for: Structure and Function of the Campylobacter jejuni Chromosome Replication Origin
Source: Front Microbiol. 2018 Jul 12;9:1533. doi: 10.3389/fmicb.2018.01533 (PMC6052347; doi:10.3389/fmicb.2018.01533)
Supplement: Supplementary file 1 [file Table_1.docx]

**Supporting Information**

**Structure and function of the *Campylobacter jejuni* chromosome replication origin**

Pawel Jaworski, Rafal Donczew, Thorsten Mielke, Christoph Weigel, Kerstin Stingl, Anna Zawilak-Pawlik

**Table S1. Strains, plasmids and proteins used in this work**

| **Strain/plasmid/ protein** | **Genotype/feature** | **Reference/ source** |
| --- | --- | --- |
| **Strains** | | |
| *E. coli* | | |
| DH5α | supE44, hsdR17, recA1, endA1, gyrA1, gyrA96, thi-1, relA1 | Laboratory stock |
| BL21 | B F- dcm ompT hsdS(rB-mB)gal | GE Healthcare |
| S17-1 | TpR SmR recA, thi, pro, hsdR-M+RP4: 2-Tc:Mu: Km Tn7 λpir | (Simon et al., 1983) |
| TOP10 | mcrA, Δ(mrr-hsdRMS-mcrBC), Phi80lacZ(del)M15, ΔlacX74, deoR, recA1, araD139, Δ(ara-leu)7697, galU, galK, rpsL(SmR), endA1, nupG | Laboratory stock |
| *C. jejuni* | | |
| 81116 | Parental strain | (Pearson et al., 2007) |
| 81-176 | Parental strain | (Hofreuter et al., 2006) |
| **Plasmids** | | |
| pOC170 | Cloning plasmid carrying the *E. coli oriC* sequence, the replication origin of pBR322 and the *bla* gene of pBR322 | (Messer et al., 1992) |
| pOC_IGR1 | A pOC170 derivative, lacking *E. coli oriC,* containing *C. jejuni* IGR1 region amplified with primers A7 and A8 and cloned between EcoRI and PstI sites | This work |
| pOC_IGR2 | A pOC170 derivative, lacking *E. coli oriC,* containing *C. jejuni* IGR2 region amplified with primers and A5 and A6 and cloned between EcoRI and PstI sites | This work |
| pOC_IGR3 | A pOC170 derivative, lacking *E. coli oriC,* containing *C. jejuni* IGR3 region amplified with primers A1 and A2 and cloned between EcoRI and PstI sites | This work |
| pOC_IGR4 | A pOC170 derivative, lacking *E. coli oriC,* containing *C. jejuni* IGR4 region amplified with primers A3 and A4 and cloned between EcoRI and PstI sites | This work |
| pOC_24 | A pOC170 derivative, lacking *E. coli oriC,* containing *C. jejuni* IGR2*-ruvC-*IGR3*-dnaA-*IGR4 region amplified with primers A4 and A5 and cloned between EcoRI and PstI sites | This work |
| pET21a(+) | IPTG inducible *E. coli* expression vector | Novagen |
| pET21b(+) | IPTG inducible *E. coli* expression vector | Novagen |
| pET21ΔT7 | A pET21b(+) derivative, modified by insertion of hybridized B6 and B7 oligonucleotides between XbaI and BamHI sites (Table S2). The inserted linker removed the T7t tag sequence and modified the reading frame of the genes cloned into the BamHI restriction site. | This work |
| pET21Strep | A pET21ΔT7 derivative, modified by insertion of hybridized B8 and B9 oligonucleotides between XhoI and SalI sites (Table S2). The linker inserted the sequences encoding linker (SA), Strep-tag (WSHPQFEK) and the stop codon. | This work |
| pET28CjDnaA | A pET28a derivative containing *C. jejuni dnaA* gene amplified with primers B1 and B2 and cloned between BamHI and XhoI sites | This work |
| pET21CjDnaA | A pET21 derivative containing *C. jejuni dnaA* gene amplified with primers B1 and B3 and cloned between BamHI and XhoI sites | This work |
| pET28Cj1509 | A pET28a derivative containing *C. jejuni Cj1509* gene amplified with primers B4 and B5 and cloned between BamHI and SalI sites | This work |
| pRY107 | Shuttle vector carrying the *E. coli* and *Campylobacter* replicon, oriT and *aph* gene | (Yao et al., 1993) |
| pRY107d | A pRY107 derivative lacking *Campylobacter oriV* | This work |
| pRY1 | A pRY107 derivative lacking *Campylobacter oriV*, containing *C. jejuni* IGR1 region amplified with primers A7 and A8 and cloned between EcoRI and PstI sites | This work |
| pRY2 | A pRY107 derivative lacking *Campylobacter oriV*, containing *C. jejuni* IGR2 region amplified with primers A5 and A6 and cloned between EcoRI and PstI sites | This work |
| pRY3 | A pRY107 derivative lacking *Campylobacter oriV*, containing *C. jejuni* IGR3 region amplified with primers A1 and A2 and cloned between EcoRI and PstI sites | This work |
| pRY4 | A pRY107 derivative lacking *Campylobacter oriV*, containing *C. jejuni* IGR4 region amplified with primers A3 and A4 and cloned between EcoRI and PstI sites | This work |
| pRY4_6 | A pRY107 derivative lacking *Campylobacter oriV*, containing *C. jejuni* IGR4 region and part of *dnaA* gene amplified with primers A4 and A14 and cloned between EcoRI and PstI sites | This work |
| pTZ57R/T | Linearized pTZ57R vector with 3'-ddT overhangs for TA cloning of PCR products with blue/white screening. | Thermo Fisher Scientific |
| pTZ_NC | A pTZ57R/T derivative, containing *S. coelicolor* DNA amplified with primers D1 and D2 and cloned between 3'-ddT overhangs |  |
| **Proteins** | | |
| 6HisCjDnaA | Recombinant, His-tagged *C. jejuni* DnaA protein | This work |
| StrepCjDnaA | Recombinant, Strep-tagged *C. jejuni* DnaA protein | This work |
| 6HisCj1509 | Recombinant, His-tagged *C. jejuni* Cj1509 protein | This work |

**References:**

Hofreuter, D., Tsai, J., Watson, R. O., Novik, V., Altman, B., Benitez, M., et al. (2006). Unique features of a highly pathogenic Campylobacter jejuni strain. *Infect. Immun.* 74, 4694–4707. doi:10.1128/IAI.00210-06.

Messer, W., Hartmann-Kühlein, H., Langer, U., Mahlow, E., Roth, A., Schaper, S., et al. (1992). The complex for replication initiation of Escherichia coli. *Chromosoma* 102, S1-6.

Pearson, B. M., Gaskin, D. J. H., Segers, R. P. A. M., Wells, J. M., Nuijten, P. J. M., and Vliet, A. H. M. van (2007). The Complete Genome Sequence of Campylobacter jejuni Strain 81116 (NCTC11828). *J. Bacteriol.* 189, 8402–8403. doi:10.1128/JB.01404-07.

Simon, R., Priefer, U., and Pühler, A. (1983). A Broad Host Range Mobilization System for In Vivo Genetic Engineering: Transposon Mutagenesis in Gram Negative Bacteria. *Nat Biotech* 1, 784–791. doi:10.1038/nbt1183-784.

Yao, R., Alm, R. A., Trust, T. J., and Guerry, P. (1993). Construction of new Campylobacter cloning vectors and a new mutational cat cassette. *Gene* 130, 127–130.
