# Supplementary material for: Structure and Function of the Campylobacter jejuni Chromosome Replication Origin
Source: Front Microbiol. 2018 Jul 12;9:1533. doi: 10.3389/fmicb.2018.01533 (PMC6052347; doi:10.3389/fmicb.2018.01533)
Supplement: Supplementary file 2 [file Table_2.DOCX]

**Supporting Information**

**Structure and function of the *Campylobacter jejuni* chromosome replication origin**

Pawel Jaworski, Rafal Donczew, Thorsten Mielke, Christoph Weigel, Kerstin Stingl, Anna Zawilak-Pawlik

**Table S2. Primers used in this work**

| **Name** | **5’ – 3’ sequence** | | **application** | |
| --- | --- | --- | --- | --- |
| **pOC and pRY plasmids** | | | See Materials and Methods | |
| A1 | GCGAATTCGGATCTATACCTAAAATTTTC | |  | |
| A2 | GCCTGCAGCGTATTCGTTTTCACTTAATT | |  |  |
| A3 | GCGAATTCGGTTCTTTAAAAGCAAAAATC | |  |  |
| A4 | GCCTGCAGGAGAAGTAATGGTGCTTGA | |  |  |
| A5 | GCGAATTCGCTTTAAAAAATTTAACGCTTT | |  |  |
| A6 | GCCTGCAGGCAACTAAAGAACAAGTTGC | |  |  |
| A7 | GCGAATTCGATTTCCTTTTCACCATAAC | |  |  |
| A8 | GCCTGCAGCTAATCCAGCTTAAACAATAA | |  |  |
| **pET plasmids** | | | See Materials and Methods | |
| B1 | CGGGATCCATGAATCCAAATCAAATACTTG | |  | |
| B2 | CCGCTCGAGTTAACTTTGACTTTTAACAAGAA | |  |  |
| B3 | CCGCTCGAGACTTTGACTTTTAACAAGAATTTTG | |  |  |
| B4 | CGGGATCCATGAAAGTTTTAATTATTGAAAATG | |  |  |
| B5 | CCGGTCGACTTATTTTTTCTTGCTGATTTCATA | |  |  |
| B6 | CTAGAAATAATTTTGTTTAACTTTAAGAAGGAGATATAG | |  |  |
| B7 | GATCCTATATCTCCTTCTTAAAGTTAAACAAAATTATT | |  |  |
| B8 | TCGAGAGCGCTTGGAGCCACCCGCAGTTCGAAAAATAAG | |  |  |
| B9 | CTCGCGAACCTCGGTGGGCGTCAAGCTTTTTATTCAGCT | |  |  |
| **pTZ57R/T plasmid** | | | See Materials and Methods | |
| D1 | GAACGCGGGACTCACCG |  | |  |
| D2 | GGATCCGACCTGAGGGTGTT |  | |  |
| **Primer extension** | | |  | |
| C1 | CTAGAGTGTGAAAAAAAGAAAT | | Fig. 2B, 3A and 5B | |
| C2 | CTAAAGTATTTTTATTGATACTT | | Fig.2B | |
| C3 | GTGAAATTCTTCAAAGCGAAA | | Fig.3B | |
| C4 | CCTTTGGATATCACAGATGC | | Fig.3E | |
| C5 | CACAAAAGCCCAAAGCTTC | | Fig.3F | |
| C6 | GCAAGACGCATTGTGATTTA | | Fig.3C | |
| C7 | ATGATTTGTCCATCGTTATTTT | | Fig.3D | |
| **EMSA** | | | See Materials and Methods | |
| E1 | IRD800-GGAGTAAGAATAGCTTCGAAT | |  | |
| E2 | IRD800-CATCGATAGGATATCCTGGG | |  |  |
| E3 | [IRD800]-ATGCAGGCCTCTGCA | |  |  |
| E4 | TCGGTACCTCGCGAA | |  |  |
| E5 | FAM-GGAGTAAGAATAGCTTCGAAT | |  |  |
| E6 | FAM-CATCGATAGGATATCCTGGG | |  |  |
| E7 | FAM-GTGCCCTGGTCTGG | |  | |
| E8 | TTTGAAGAATTTCACAATTTCAACA | |  | |
| E9 | TGTTGAAATTGTGAAATTCTTCAAACCAGACCAGGGCAC | |  | |
| E10 | TTTGAAGAATGTTACAATTTCAACA | |  | |
| E11 | TGTTGAAATTGTAACATTCTTCAAACCAGACCAGGGCAC | |  | |
| E12 | TTTGAAGAAAGTATCAATTTCAACA | |  | |
| E13 | TGTTGAAATTGATACTTTCTTCAAACCAGACCAGGGCAC | |  | |
| **Plasmid maintenance** | | | See Materials and Methods | |
| F1 | CGGGATCCATGAAAAACGTTGGCGACCTG | |  | |
| F2 | CCGCTCGAGTTAATACTCTTTACCTGTTACCC | |  |  |
| F3 | ATCGCAACTTGATGTGCTTG | |  |  |
| F4 | ACCACTTGATTCTACGCGG | |  |  |
| M13 | GTAAAACGACGGCCAGT | |  |  |
| rM13 | CAGGAAACAGCTATGAC | |  |  |
