# Supplementary material for: Structure and Function of the Campylobacter jejuni Chromosome Replication Origin
Source: Front Microbiol. 2018 Jul 12;9:1533. doi: 10.3389/fmicb.2018.01533 (PMC6052347; doi:10.3389/fmicb.2018.01533)
Supplement: Supplementary file 3 [file Image_1.PDF]

	ttattttggcactaccatatcgctacaacattctatcaccgtatgtacatttccacgcgggttaaaatcccccacaactt		80
	taatccattttggttttaatttatcttttaaagtattataaatttcattgatacttgcttcatgtgaaacatttctatac		160
queF	ataaaagtattgatataaagttttatcgccttaagctcaaccacaaatttatcaggcatatactcaagataaatcgtcgc		240
	aaagtcaggataacccgaacgcggacaacaacacataaattcaggtaaagtgatttttaatgatatagtcatttttggcat		320
	catttggccaaatttccatattttctacatcaaattctttgatttccttttcaccataacgcataaatttccttaaaatt		400
	taaattcgaacaaaaattataacaaaataagtttttaaaacgattatgatacagataagaaaatgcaattttattgcaac		480
	tcaccccctagcaagagtgagttagcaataaattgaaggcagttcaagcctaagcctagtattacaagactaccaagata	IGR1	560
	ggcttgtaaagcaaggctgctccctttaagggagcaaaaaacaatattttatagaatactaagcttaaattattgtttaa		640
	gctggattagagtttgaagcatttgatcactcgttgaaatggtttttgagtttgcttgataacctctttgaatgataata		720
	agctctgttaaagaacgacttaaatccacatttgacatttcaagagctgaggttttcatctcaccacgacctcctgttcc		800
	tgcttcacctactacgatattaccactatttgcagtgactttaaaaagatttccaccaatttcctcaagacctgagttat		880
	ttgccactgaagccattgctatttttgctacagcaaaggttttaccattagtaaattcacctaagatattacctttgtca		960
	tccacacggatagcatcaggttttaagttacctgaagtataaccatcggttgcctgtcctgttagagttgaagcagaatt		1040
	tgagcttacaaggccgtcattgcttccacttgttccaaaggaaagtttgatttgttgatttggtgcggcaccattgtttg		1120
	gtgaaaaatttatcgtttttggtgtataattagctaaagatccatcattattaaatcttgctgttcctacgatgatattt		1200
	gtaggaccttcgcctgtagtgttaatctctgcaggttcaggtacacggatgatcatttgccattcattacccccatcttg		1280
	agtggtgctttgcttaacaaactgcacttctaaagtgtgttttgaacctaaagaatcgtaaatttcaagtccagctgaaa		1360
	aagcagaaagcttaagttgctcgctttctttgatctgatttcctataaccaaaggaccatcgaaagctttaaaaatttta		1440
	gtaaaagcatcattagtacttacagttccttgtttattactataagcagttatattaaaactcatattttttggatttgt		1520
	tgtagcagcaccagcaccagcaccagcattagctggtatagtagaagtttcatttgcgttagaaatagcaaaattaccat		1600
	tttcagttaccactactttacagcttgattgatatcattagcgtcaaatgttggagtagcattatctattataccacta		1680
	ccattatagtcaaccccatacctagcatctctttgcaaaagctctcttaaatcttctgtggttctaaaagttctttcagt		1760
	agtattttttaaactcccttgtatagcatcccaataattagcagaagcgggatcggttggtctattaccgttattaggct		1840
flgE	gaaacgccggtccaccatcaggatttatcataggtggtatagtaacaggatttgaactataaatatacttatgtgctgtc		1920
	actactttaacatttgtggttgacctgcttgagctgctccagctatccaatcagtatcattctgagcagttgttagatt		2000
	accaccttgaggagaaaaaacaccagtattagcattataattaatagtatttctttctcctgcagaattcccaacattaa		2080
	ctgttaaatcgatatttttcatattatctgttgtaccatcggcattattattgacaaattctatacctgaaccatctgct		2160
	ttttttaccgctttactcctgttccatctcttgtatcagttggtgcagtaaaggtattgatataagctatagcttcatc		2240
	taagcttcttatattatcattttgaattcttactccatttaaattaatatccaaagttacagctatatctttattacccc		2320
	agaaaataacattatttgttgtgctactgttaaattaggatcaaagacatttgcaccattagctctatctgtagtaaat		2400
	ttagcatctgcataagatacccaaatcccttgtccatctctaagatttaatcctgtgccattagcattaaataaagctcc		2480
	agcatctacaccctttcagtcacttctacagaattcttagaagtcgtataaaactgagtggttcctgtatcgttttcat		2560
	cttctgctctttgggttttagtattccatccatgaacagaatcaagtgcataaagatttctacttgaagttcctatattt		2640
	aaaccgctatttaaattcgctttgatagcaacttcagtagattttgctgcagggatatgcataccgggatcgataaaaat		2720
	atttgtggagttcttgaactatctatagtttgatcatcccaattgatattccacccttggacaacaaaacctgcattat		2800
	taacaaaatttccataagcatcgagtttaaaatccccgctccttgtaagatagtttgtaagaccaccatcatcactcacc		2880
	ataaaaaaaccatcaccatttatagcaacatcagtgtttttatctgtggtttgaactgaaccttgagaatgaattctagt		2960
	tgtagaacttactgaaacaccaagaccgatttgaagtggattagatccgcctcttccatcagttggagctgtagcgattt		3040
	tcacagtttggctaaacatagtcccaaaatctgcacgagagtatttaaaaccagtggtattaacatttgaaatgttatta		3120
	ccttcaacatccatcgcaacttgatgtgcttgtagtccgcttacgccagaccaaagtgatctcatcatcttaaatccttt		3200
	ataaaatattgctttaaaaaatttaacgctttggctttaaaaaactttgcgtttatgtgaagtctacaagcaagttctg	IGR2	3280
	ttccaacttttgaattttttatgaattttttaaaaatttatagaaaaaatttaaaggtattttaggagtttatgaagct		3360
	ttgggcttttgtgtaataaaaattactaaaatcacaccctaactcttaaatttgctgcatgggttaaagctactgcgata		3440
	gcatctgtgatatccaaaggcttaatatctttactcagtcctaaaagcctttttaccataaaagcaacttgttctttagt		3520
	tgctttggcttttcctgtaacggctttttttacttgtaagggagtgtattctgcaaaatctccatgaatttgaagaattt		3600
ruvC	ttaaagatagggctccgcgaaattgtgcgagtttaaaacggtttttggattgtaagcaaaaaagatatcttctatagcc		3680
	acctcatcaaaagaatgatttttaaaaatcaagtccaaaccctcgcaaagctctgtgatttggtattgtaaggtatttgg		3760
	ctttatctttataagtcctgcttctatgagaatgtttttcctttgttggcttctatgatagcataaccgcaatttcttg		3840
	aaccaggatctatacctaaaatttcaatcttccaccttaatcaataatgaaatgcaaaagcttgattttaacctttttt		3920
	tataaaatttaagataaaatttataagcttacaaagactaaagagagattatgaatccaaatcaaatacttgaaattta	IGR3	4000
	aaaaaagaattaagtgaaaacgaatacgaaaattatatcgctatcttaaaatttaacgaaaaacaaagcaaagcagattt		4080
	tctagtctttaacgctcctaatgagcttttagccaaattcatacaaacaaaatacggtaaaaaaatttcacatttttatg		4160
	aagtacaaagcggaaataaagcgagcgttttgatacaagcacaaagtgctaaacaaagtagcaaaagcactaaaatcgat		4240
	atcgctcatatcaaggcgcaaagtacgatttaaatccttcttttacttttgaaagctttgtagtggggattctaacaa		4320
	atacgcttatggagcttgtaaagctatctcacaaaaagacaaactgggaaaactttataatcctatctttatctatgggc		4400
	ctacagggcttggaaaaacgcacttgcttcaagctgtgggaaatgcaagtttggaaatgggaaaaaaagtgatttatgct		4480
	acgagtgaaaattttatcaatgatttacttcaaatttaaaaaatggctctttagataaatttcacgaaaaatatagaaa		4560
	ttgtgatgttttactcatagatgatgtgcagttttaggaaaaaccgataaaattcaagaagaatttttctttatattta		4640
dnaA	atgaaatcaaaaataacgatggacaaatcatcatgacttcagacaatccacccaacatgctaaaaggtataaccgaacgc		4720
	ttaaaaagtcgtttgcacatgggatcatagctgatataactccacctcaactagatacaaaaatagccatcataagaaa		4800
	aaaatgtgaatttaacgatatcaatctttctaatgatatcataaactatatcgctacttctttaggggataatataagag		4880
	aaatcgaaggtatcatcataagtttaaatgcttatgcaaccatactaggacaagaaatcacactcgaacttgccaaaagt		4960
	gtgatgaaagatcatatcaaagaaaagaaagaaaatatcactatagatgacatttatctttggtatgtaaagaatttaa		5040
	catcaaaccaagcgatgtgaaatccaataaaaaaactcaaaatatagtcacagcaagacgcattgtgatttacctagcta		5120
	gggcacttacggctttgactatgccacaacttgcgaattatttgaaatgaaagatcatacagctatttcacataatgtt		5200
	aaaaaaatcacagaaatgatagaaaatgatggttctttaaaagcaaaaatcgaagaacttaaaaacaaaattcttgttaa		5280
	aagtcaaagttaagtgaaaggatgtgaaaaataaattctagagtgtgaaaaaaagaaattaagcaaagtatgataaaata		5360
	caaatttgattatttcgctttgaagaatttcacaatttcaacaagcttattgttacaacgaatttaaaattaaaataaac	IGR4	5440
	caaggagaaaaaatgaagttaagtatcaataaaaatactttagaatctgcagtgattttatgtaatgcttatgtagaaaa		5520
	aaaagactcaagcaccattacttctcatctttttttttcatgctgatgaagataaacttcttattaaagctagtgattatg		5600
	aaataggtatcaactataaaataaaaaaaatccgcgtagaatcaagtggttttgctactgcaaatgcaaaaagtattgca		5680
	gatgttattaaaagcttaaacaatgaagaagttgttttagaaaccattgataattttttatttataagacaaaaaagcac		5760
	aaaatacaaacttcctatgtttaatcatgaagattttccaaattttccaaatacagaaggaaaaaaccaatttgacattg		5840
	attcaagtgatttaagccgttctcttaaaaagatattaccaagtattgatacaaataacccaaaatactccttaaatggt		5920
dnaN	gcatttttagatataaaaacagataaaattaacttcgtaggaactgatacaaaacgccttgcaatctatactttagaaaa		6000
	agcaaataatcaagaatttagttttagtatccctaaaaaagctattatggaaatgcaaaaacttttctatgaaaaaatag		6080
	aaattttttatgatcaaaatatgcttattgccaaaaatgaaaattttgaattctttacaaaacttattaacgataaattt		6160
	ccagattatgaaaaagttataccaaaaactttcaaacaagaactcagtttttcaactgaagattttatagatagtcttaa		6240
	aaaaatcagtgttgtaactgaaaaaatgaaacttcattttaacaaagataaaatcatctttgaaggtataagtttagaca		6320
	atatggaagcaaaaacagaacttgaaattcaaacaggagtaagtgaagaatttaatcttactataaaaatcaaatattta		6400
	cttgatttcttaacttctatagaagaagaaaaattcactttaagtgtaaatgaacctaattcagcatttatagtcaaatc		6480
	ccaaggactatcaatgattatcatgcctatgattttgtaa		

predicted DnaA boxes; *genes* (fragments); predicted DUE; — degenerated DnaA-trio

Figure S1. *In silico* analysis of the *C. jejuni* 81116 *queF-dnaN* region. For detailed description see Main text and Figure 1.
