# Supplementary material for: Structure and Function of the Campylobacter jejuni Chromosome Replication Origin
Source: Front Microbiol. 2018 Jul 12;9:1533. doi: 10.3389/fmicb.2018.01533 (PMC6052347; doi:10.3389/fmicb.2018.01533)
Supplement: Supplementary file 4 [file Image_2.PDF]

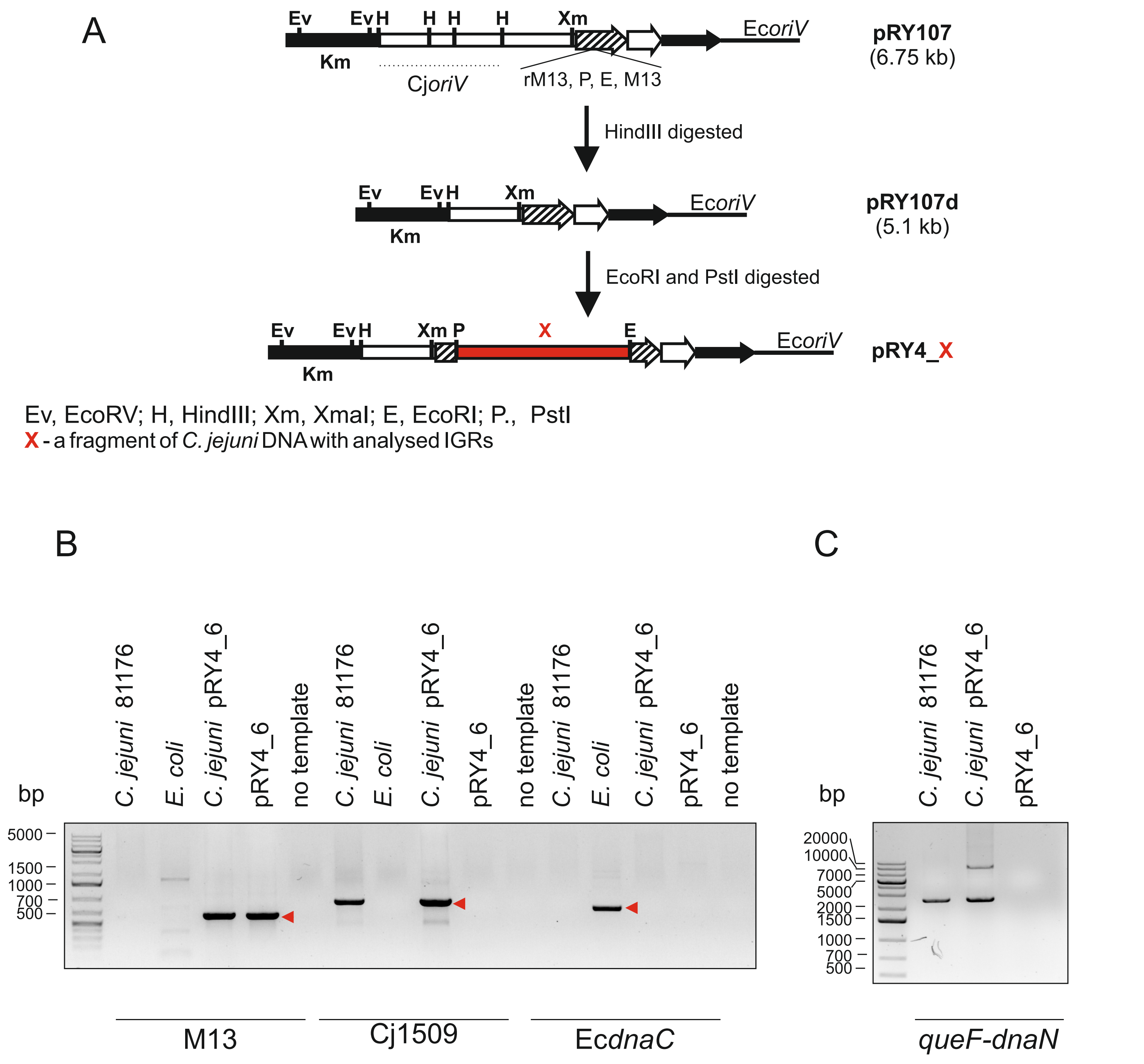

Figure S2. Identification of the *oriC* region sufficient for *in vivo* plasmid replication in *C. jejuni*. **A.** Schematic representation of pRY107 derivatives construction. The name and approximate size of the plasmid are indicated on the right, designations are identical to Yao et al.,1993 **B.** PCR analysis of *C. jejuni* genomic DNA isolated after conjugative plasmid transfer using primer pairs (Supplementary Table S2), which amplify plasmid insert (M13, rM13, expected size of the PCR product: 538 bp), *C. jejuni Cj1509* gene (B4-B5, expected size of the PCR product: 900 bp) or *E. coli dnaC* (F1-F2, expected size of the PCR product: 760 bp)*;* the latter PCR was done to exlude the possibility of contamination of *C. jejuni* genomic DNA by *E. coli* DNA. *C. jejuni* 81176 wild type strain, *E. coli* genomic DNA and te pRY_6 plasmid are included as control templates. Specifically amplified PCR products are indicated by red arrowheads. **C.** PCR analysis of genomic DNA isolated from *C. jejuni* pRY4_6 conjugants. A primer pair F3-F4 was used (Supplementary Table S2), which amplifies the chromosomal *queF-dnaN* region (Figure 1) . The 2507 bp PCR product indicates for the presence of the intact *queF-dnaN* region, as in the wild type strain, while the higher molecular weight PCR product of approx. size 8000 bp, indicates for an integration of pRY4_6 plasmid into the *C. jejuni* chromosome via single crossing-over. The lower intensity of high molecular weight PCR product in comparison to 2507 bp PCR product is probably caused by the lower efficiency of amplification of longer DNA fragments by a polymerase.
