# Supplementary material for: Structure and Function of the Campylobacter jejuni Chromosome Replication Origin
Source: Front Microbiol. 2018 Jul 12;9:1533. doi: 10.3389/fmicb.2018.01533 (PMC6052347; doi:10.3389/fmicb.2018.01533)
Supplement: Supplementary file 5 [file Image_3.PDF]

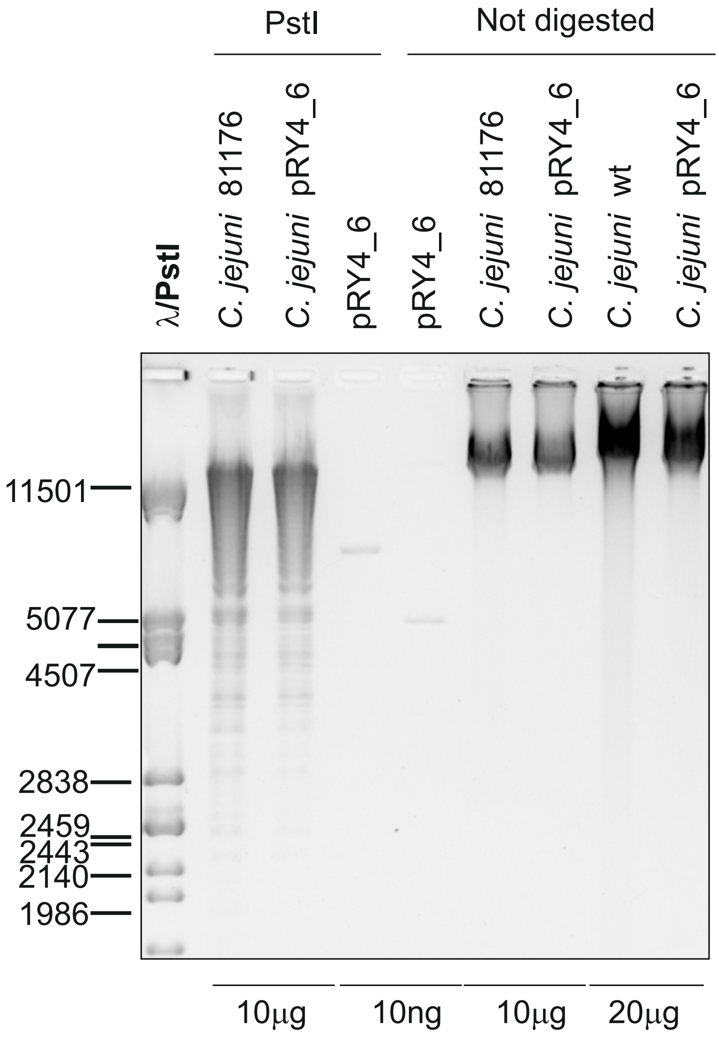

Figure S3. The picture of DNA resolved in an agarose gel. *C. jejuni* genomic DNA (81176 wild type strain and pRY4_6 conjugant strain) and the pRY4_6 plasmid, undigested or digested with PstI, were resolved in 1% agarose gel. DNA was subsequently transfered onto a nylon membrane. The results of the probe hybridisation to the membrane-bound DNA is presented on Figure 2.
