# Supplementary material for: Structure and Function of the Campylobacter jejuni Chromosome Replication Origin
Source: Front Microbiol. 2018 Jul 12;9:1533. doi: 10.3389/fmicb.2018.01533 (PMC6052347; doi:10.3389/fmicb.2018.01533)
Supplement: Supplementary file 7 [file Image_5.PDF]

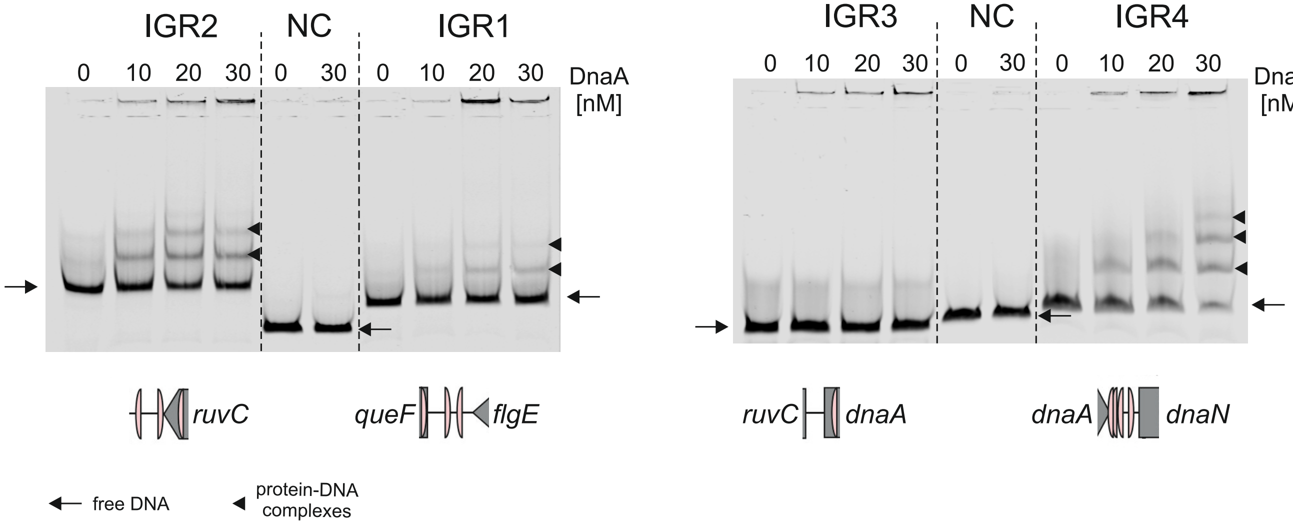

Figure S5. DnaA protein interaction with intergenic regions. The gel-retardation assay was performed using DNA fragments (IRD-labeled IGR1, IGR2, IGR3, IGR4 and negative control (NC) (Supplementary Materials) that were incubated with the indicated amounts of 6His-tagged *C. jejuni* DnaA protein. The nucleoprotein complexes were resolved on a 4% polyacrylamide gel. The number of observed DnaA-DNA complexes depended on the number, arrangement and quality (high- or low-affinity) of DnaA boxes. Please note, that DnaA boxes preseneted on schemes were predicted *in sillico* and may differ from sequences bound by DnaA *in vitro*.
