# Supplementary material for: Structure and Function of the Campylobacter jejuni Chromosome Replication Origin
Source: Front Microbiol. 2018 Jul 12;9:1533. doi: 10.3389/fmicb.2018.01533 (PMC6052347; doi:10.3389/fmicb.2018.01533)
Supplement: Supplementary file 8 [file Image_6.PDF]

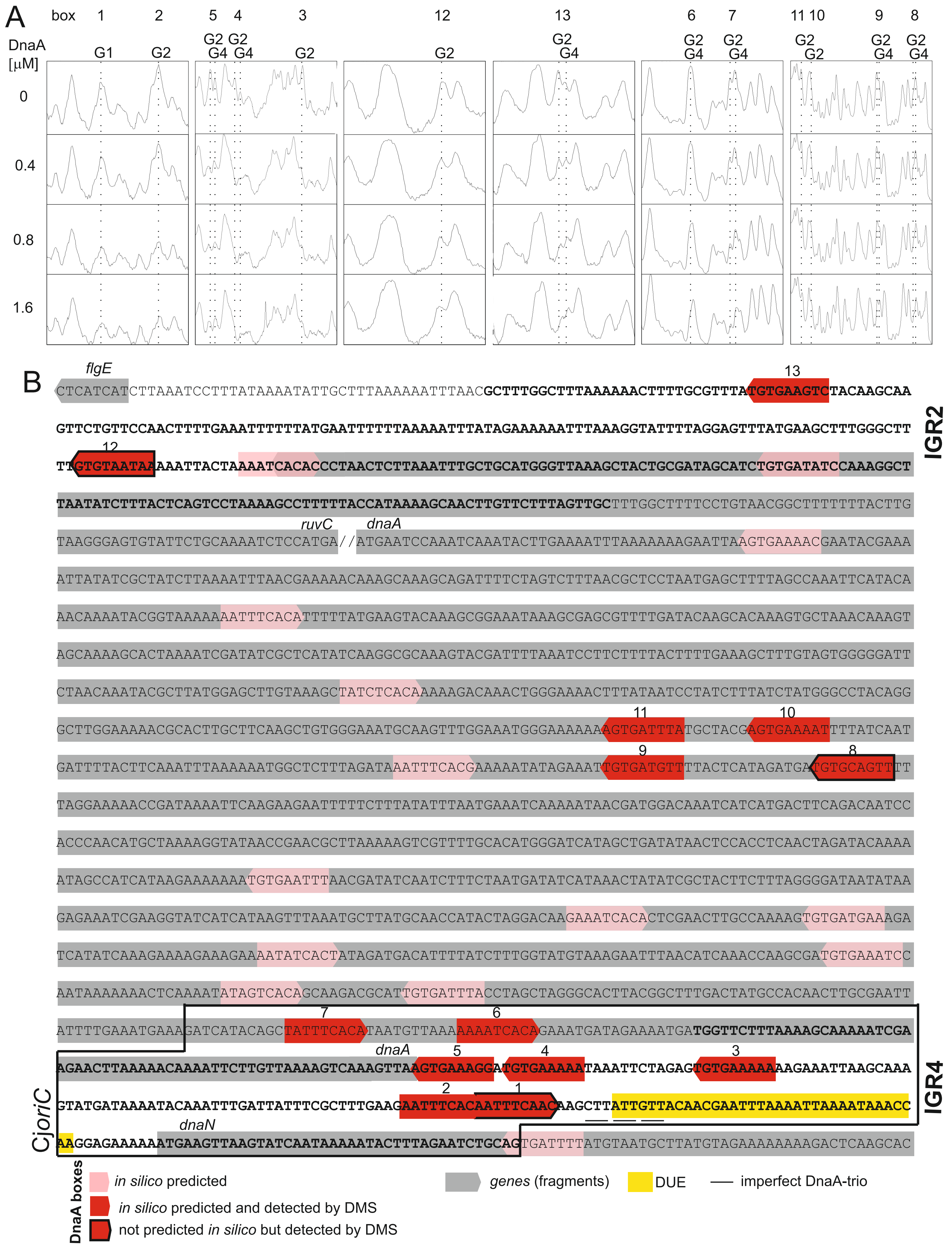

Figure S6. Identification of the DnaA boxes localized in the IGR2, IGR4 and Cj*dnaA* gene. **A.** Densitometric analysis of DMS footprinting gels. The concentrations indicated next to the plots correspond to the analyzed lanes in Figure 4 A-F. Protected guanosine residues (G) with the position of protection for each DnaA box are indicated above the plots and with dotted lines. **B.** The structure of the *C. jejuni* origin of replication and localization of *in vitro*-identified DnaA boxes in the *dnaA* gene. The discontinuousness of the DNA sequence is indicated by a double slash. The sequence that sustained self-replication of a minichromosome is boxed, while IGR2 and IGR4 sequences are in bold. The *C. jejuni* DnaA boxes identified experimentally by DMS footprinting (in red) and putative boxes identified *in silico* based on Epsilonproteobacterial consensus sequence (in pink) are marked.
