# Supplementary material for: Structure and Function of the Campylobacter jejuni Chromosome Replication Origin
Source: Front Microbiol. 2018 Jul 12;9:1533. doi: 10.3389/fmicb.2018.01533 (PMC6052347; doi:10.3389/fmicb.2018.01533)
Supplement: Supplementary file 9 [file Image_7.PDF]

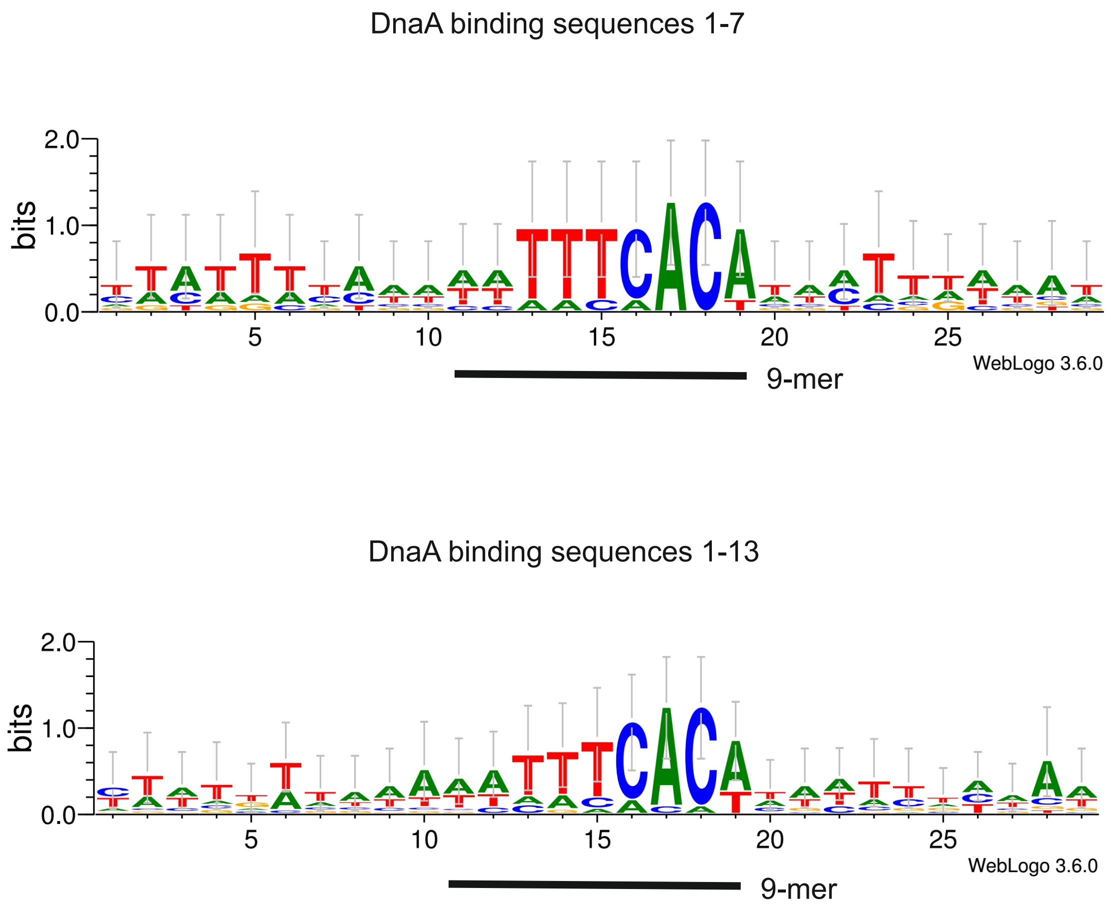

Figure S7. *C. jejuni* DnaA binding sites sequence alignment. Aligned sequences (29 nt) contained DMS identified putative 9-mer DnaA boxes 1-7 (top panel) or 1-13 (bottom panel), extended upstream and downstream by 10 nt.
