# Supplementary material for: Structure and Function of the Campylobacter jejuni Chromosome Replication Origin
Source: Front Microbiol. 2018 Jul 12;9:1533. doi: 10.3389/fmicb.2018.01533 (PMC6052347; doi:10.3389/fmicb.2018.01533)
Supplement: Supplementary file 6 [file Image_4.PDF]

A

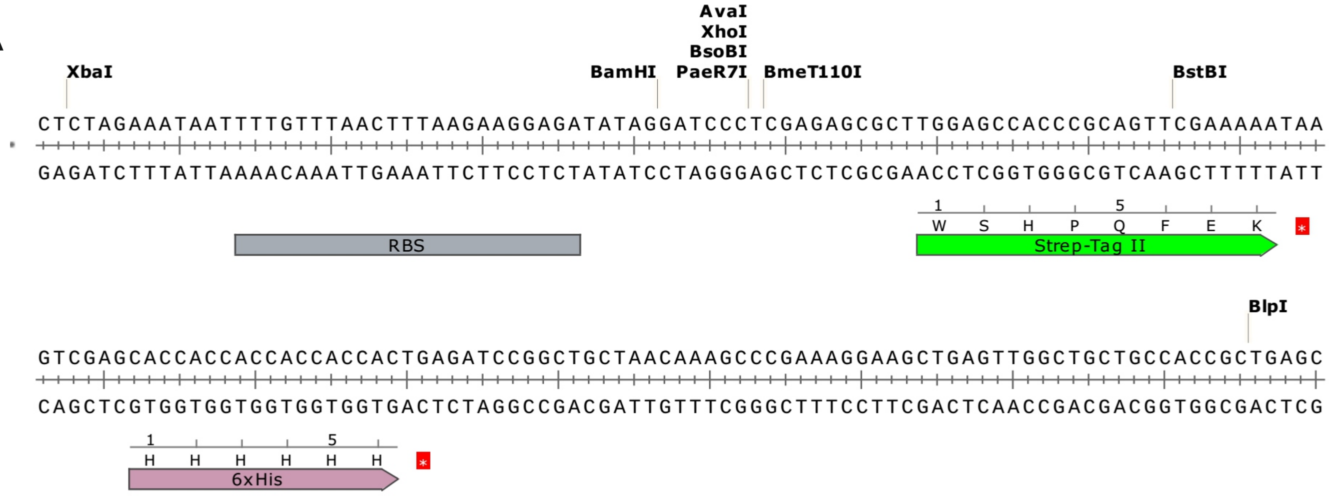

B

6HisCjDnaA
MGSSHHHHHHSSGLVPRGSHMASMTGGQQMGRGSMNPNQILENLKKELSENEYENYIAILKFNEKQSKADFLVFNAPNELLAKFI
QTKYGKKISHFYEVQSGNKASVLIQAQSAKQSSKSTKIDIAHIKAQSTILNPSFTFESFVVGDSNKYAYGACKAISQKDKLGKLYNPIFI
YGPTGLGKTHLLQAVGNASLEMGKKVIYATSENFINDFTSNLKNGSLDKFHEKYRNCDVLLIDDVQFLGKTDKIQEEFFFIFNEIKNND
GQIIMTSDNPPNMLKGITERLKSRFAHGIIADITPPQLDTKIAIIRKKCEFNDINLSNDIINYIATSLGDNIREIEGIIISLNAYATILGQEITLEL
AKSVMKDHIKEKKENITIDDILSLVCKEFNIKPSDVKSNKKTQNIVTARRIVIYLARALTALTMPQLANYFEMKDHTAISHNVKKITEMIEN
DGSLKAKIEELKNKILVKSQS*
StrepCjDnaA
MNPNQILENLKKELSENEYENYIAILKFNEKQSKADFLVFNAPNELLAKFIQTKYGKKISHFYEVQSGNKASVLIQAQSAKQSSKSTKI
DIAHIKAQSTILNPSFTFESFVVGDSNKYAYGACKAISQKDKLGKLYNPIFIYGPTGLGKTHLLQAVGNASLEMGKKVIYATSENFINDF
TSNLKNGSLDKFHEKYRNCDVLLIDDVQFLGKTDKIQEEFFFIFNEIKNNDGQIIMTSDNPPNMLKGITERLKSRFAHGIIADITPPQLD
TKIAIIRKKCEFNDINLSNDIINYIATSLGDNIREIEGIIISLNAYATILGQEITLELAKSVMKDHIKEKKENITIDDILSLVCKEFNIKPSDVKSN
KKTQNIVTARRIVIYLARALTALTMPQLANYFEMKDHTAISHNVKKITEMIENDGSLKAKIEELKNKILVKS**QSLESAWSHPQFEK***
6HisCj1509
MGSSHHHHHHSSGLVPRGSHMASMTGGQQMGRGSMKVLIIENEIYLAQSISIKLSDAGYSCEIINSFDEYNGEKYYDIVLLSTNTNN
FLKAVGQFKHSIIILLISYISTDTVSNPLKLGASDYIQKPFMIEELIRKIKHYQDFRKLSILNKAYQSYIKSRLETIKIPEYNYKKLKLPLILKS
NKQSSADAFVFNYANECDITLSFIDLTSTNSVEKVMKLPTENNLLFLSNFQALKATEKEKLLDFIQNKNVILHTNSNTDDLKINCINLND
NEKNIDSNEILTIDEYVKYVIINYQNIFPDTDLSKKLGISRKSLWEKRKKYEISKKK*

C

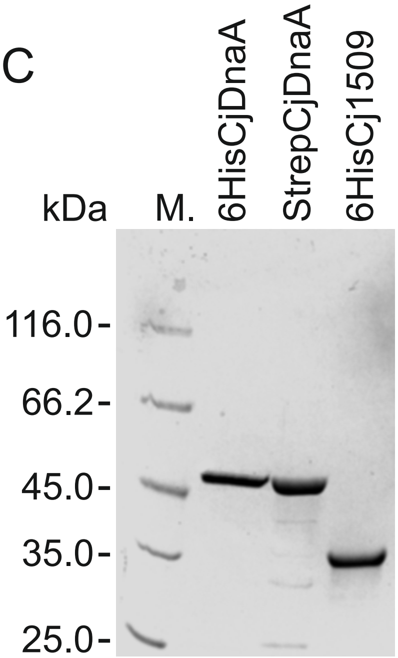

Figure S4. Recombinant proteins used to study the *C. jejuni oriC*. **A**. A scheme of a MCS site in the modified pET21Strep vector. **B**. Sequences of recombinant proteins. 6HisCjDnaA, N-terminal His-tagged CjDnaA (Mw 53.4 kDa), StrepCjDnaA, C-terminal Strep-tagged CjDnaA (Mw 51.1 kDa); 6HisCj1509; N-terminal His-tagged Cj1509 (Mw 37,8 kDa). His-tag is shown in pink, while Strep-tag is shown in green. **C.** Purification of the proteins, which were used in this study. Elutions with the highest purity and concentration were analyzed in a 10% SDS-PAGE gel (5 µg/well) and stained with Coomassie Brilliant Blue. M, molecular weight marker.
