# Supplementary material for: Structure and Function of the Campylobacter jejuni Chromosome Replication Origin
Source: Front Microbiol. 2018 Jul 12;9:1533. doi: 10.3389/fmicb.2018.01533 (PMC6052347; doi:10.3389/fmicb.2018.01533)
Supplement: Supplementary file 10 [file Image_8.PDF]

Cj1509 box probes

Cj1509box1

FAM-GTGCCCTGGTCTGGTTTGAAGAA**TTTCACA**ATTTCAACA
CACGGGACCAGACCAAACTTCTT**AAAGTGT**TAAAGTTGT

Cj1509box2

FAM-GTGCCCTGGTCTGGTTTGAAGAA**TGTTACA**ATTTCAACA
CACGGGACCAGACCAAACTTCTT**ACAATGT**TAAAGTTGT

Cj1509box3

FAM-GTGCCCTGGTCTGGTTTGAAGAA**AGTATCA**ATTTCAACA
CACGGGACCAGACCAAACTTCTT**TCATAGA**TAAAGTTGT

sequences of Cj1509 boxes

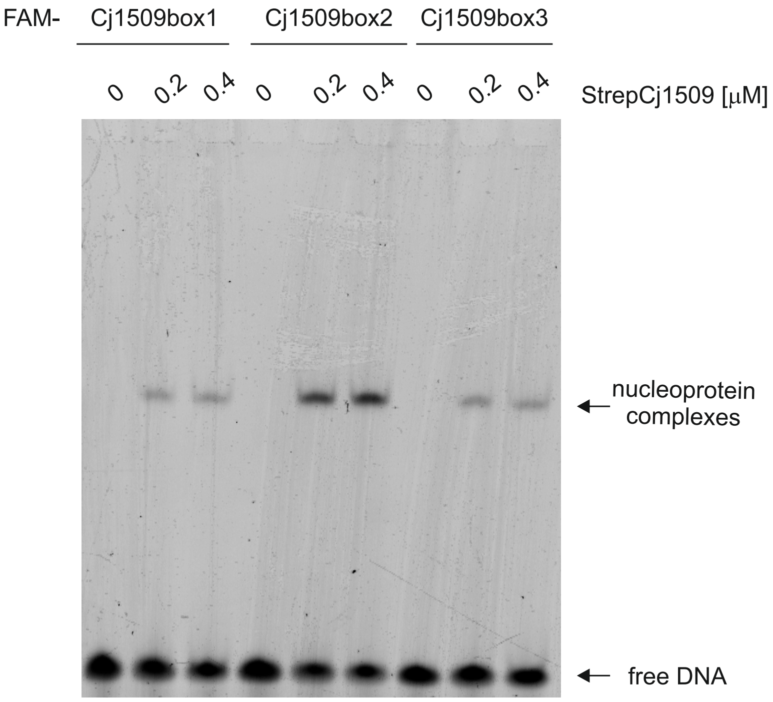

Figure S8. Cj1509 protein interaction with Cj1509 boxes. The gel-retardation assay was performed using FAM-labeled oligonucleotide probes (Cj1509box1, Cj109 box2 and Cj1509 box3), that were incubated with the indicated amounts of the recombinant *C. jejuni* 1509 protein. The nucleoprotein complexes were resolved on a 4% polyacrylamide gel.
