# Supplementary material for: Structure and Function of the Campylobacter jejuni Chromosome Replication Origin
Source: Front Microbiol. 2018 Jul 12;9:1533. doi: 10.3389/fmicb.2018.01533 (PMC6052347; doi:10.3389/fmicb.2018.01533)
Supplement: Supplementary file 11 [file Image_9.PDF]

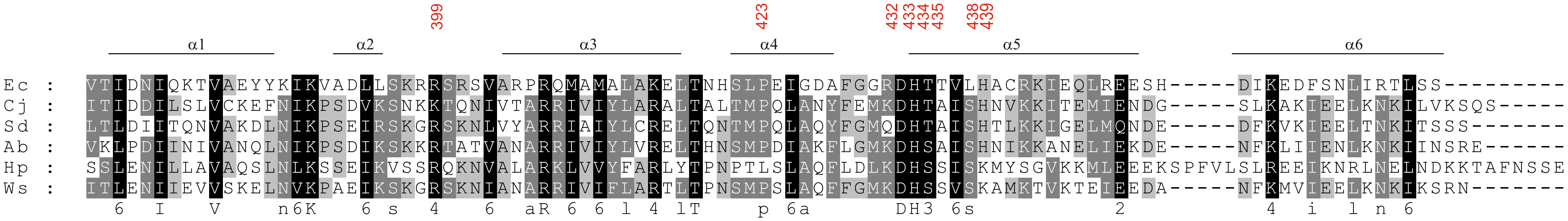

Figure S9. Multiple-sequence alignment of DnaA homologs from *E. coli* (Ec), *C jejuni* (Cj), *S. denitrificans* (Sd), *A. butzleri* (Ab), *H. pylori* (Hp) and *Wolinella succinogenes* (Ws). The red numbers indicate amino acids in *E. coli* DnaA involved in base-specific interactions.
